# Impacts of Dams and Global Warming on Fish Biodiversity in the Indo-Burma Hotspot

**DOI:** 10.1371/journal.pone.0160151

**Published:** 2016-08-17

**Authors:** Yuichi Kano, David Dudgeon, So Nam, Hiromitsu Samejima, Katsutoshi Watanabe, Chaiwut Grudpan, Jarungjit Grudpan, Wichan Magtoon, Prachya Musikasinthorn, Phuong Thanh Nguyen, Bounthob Praxaysonbath, Tomoyuki Sato, Koichi Shibukawa, Yukihiro Shimatani, Apinun Suvarnaraksha, Wataru Tanaka, Phanara Thach, Dac Dinh Tran, Tomomi Yamashita, Kenzo Utsugi

**Affiliations:** 1 Institute of Decision Science for a Sustainable Society, Kyushu University, Fukuoka, Fukuoka, Japan; 2 School of Biological Sciences, The University of Hong Kong, Hong Kong SAR, China; 3 Inland Fisheries Research and Development Institute of Fisheries Administration, Phnom Penh, Cambodia; 4 Institute for Global Environmental Strategies, Hayama, Kanagawa, Japan; 5 Department of Urban and Environmental Engineering, Kyushu University, Fukuoka, Fukuoka, Japan; 6 Department of Zoology, Kyoto University, Kyoto, Kyoto, Japan; 7 Department of Fisheries, Ubon Ratchathani University, Ubon Ratchathani, Thailand; 8 Department of Biology, Mahasakham University, Maha Sarakham, Thailand; 9 Faculty of Fisheries, Kasetsart University, Bangkok, Thailand; 10 Department of Fisheries Management and Economics, Can Tho University, Chan Tho, Vietnam; 11 Faculty of Science, National University of Laos, Vientiane, Lao PDR; 12 Siem Reap Freshwater Fishes Laboratory, Siem Reap, Cambodia; 13 Nagao Natural Environment Foundation, Sumida, Tokyo, Japan; 14 Museum of Natural and Environmental History, Shizuoka, Shizuoka, Japan; 15 Faculty of Fisheries Technology and Aquatic Resources, Maejo University, Chiang Mai, Thailand; 16 Graduate Education and Research Training Program in Decision Science for Sustainable Society, Kyushu University, Fukuoka, Fukuoka, Japan; University of Hyogo, JAPAN

## Abstract

Both hydropower dams and global warming pose threats to freshwater fish diversity. While the extent of global warming may be reduced by a shift towards energy generation by large dams in order to reduce fossil-fuel use, such dams profoundly modify riverine habitats. Furthermore, the threats posed by dams and global warming will interact: for example, dams constrain range adjustments by fishes that might compensate for warming temperatures. Evaluation of their combined or synergistic effects is thus essential for adequate assessment of the consequences of planned water-resource developments. We made projections of the responses of 363 fish species within the Indo-Burma global biodiversity hotspot to the separate and joint impacts of dams and global warming. The hotspot encompasses the Lower Mekong Basin, which is the world’s largest freshwater capture fishery. Projections for 81 dam-building scenarios revealed progressive impacts upon projected species richness, habitable area, and the proportion of threatened species as generating capacity increased. Projections from 126 global-warming scenarios included a rise in species richness, a reduction in habitable area, and an increase in the proportion of threatened species; however, there was substantial variation in the extent of these changes among warming projections. Projections from scenarios that combined the effects of dams and global warming were derived either by simply adding the two threats, or by combining them in a synergistic manner that took account of the likelihood that habitat shifts under global warming would be constrained by river fragmentation. Impacts on fish diversity under the synergistic projections were 10–20% higher than those attributable to additive scenarios, and were exacerbated as generating capacity increased—particularly if CO_2_ emissions remained high. The impacts of dams, especially those on river mainstreams, are likely to be greater, more predictable and more immediately pressing for fishes than the consequences of global warming. Limits upon dam construction should therefore be a priority action for conserving fish biodiversity in the Indo-Burma hotspot. This would minimize synergistic impacts attributable to dams plus global warming, and help ensure the continued provision of ecosystem services represented by the Lower Mekong fishery.

## Introduction

Freshwater biodiversity, especially that of fishes, is jeopardized by a range of factors globally [1–4]. This reflects the variety and extent of human activities that degrade freshwater environments generally [3], as well as factors more particular to fishes such as impacts of alien species [4] and overexploitation [5]. Dam construction directly impacts fish biodiversity and both transforms and fragments riverine habitats [2–3, 6], but brings benefits to humans in terms of water supply and electricity generation. At the same time, global warming also has profound implications for freshwater ectotherms and their conservation [7–8] and such threats can be exacerbated by complex, often synergistic, interactions between various anthropogenic threats and stressors [9]. For instance, fragmentation of river networks by dams limit the ability of fishes to adapt to warming temperatures by shifting their ranges to occupy areas upstream [10].

The Indo-Burma region is a global biodiversity hotspot [11]. It is characterized by high fish richness, although the ecology and distribution of that fauna is insufficiently known [12–13]. This is a significant shortcoming, given the importance of inland capture fisheries to regional food security and livelihoods: for instance, yields from the Lower Mekong Basin (LMB) alone are estimated at 2.1–2.2 M t annually, and it is the world’s largest freshwater fishery [14–15].

**Fig 1:**
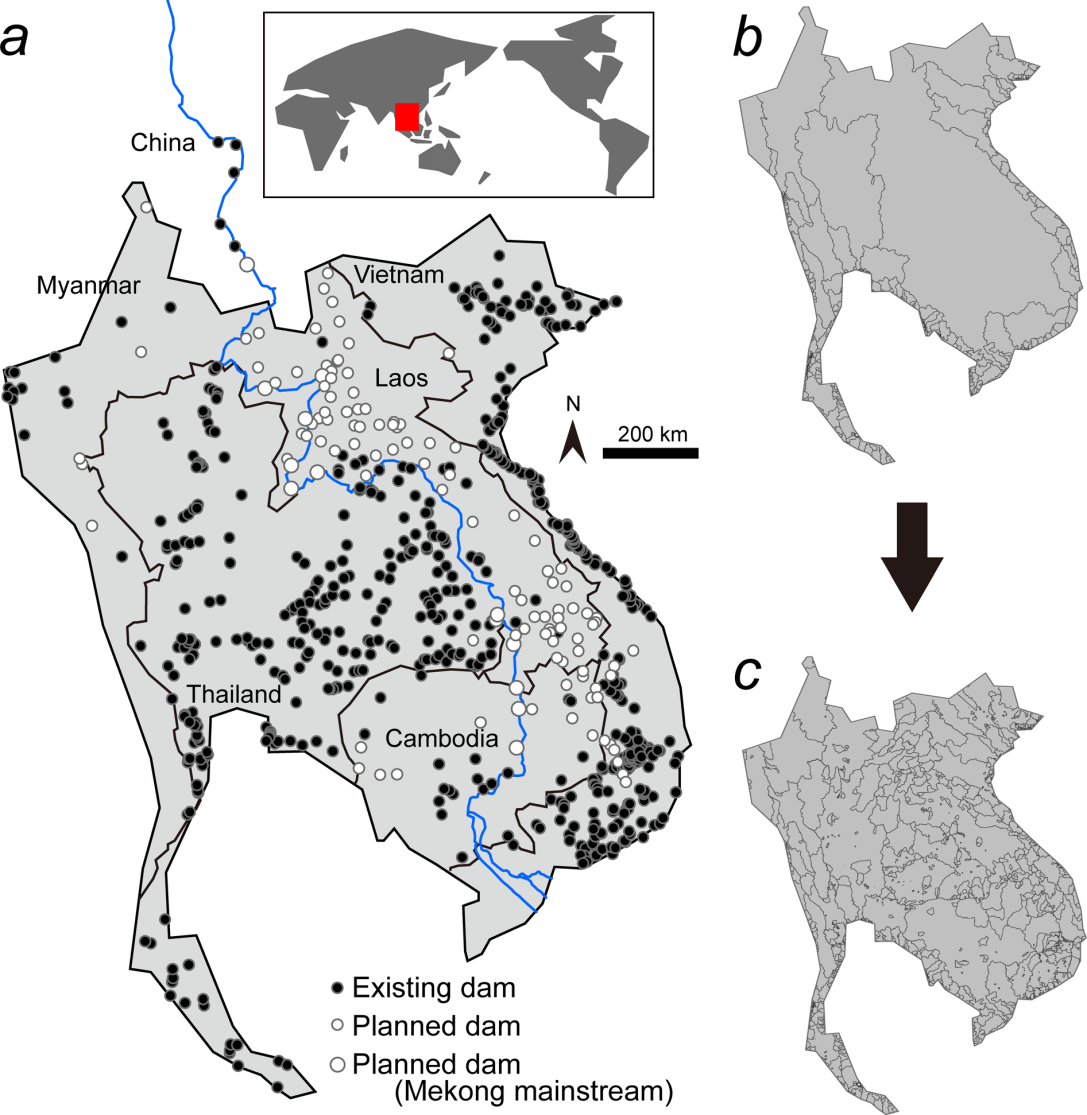
Dams and fragmentation in Indo-Burma Biodiversity Hotspot. (a) Existing (solid circles) and planned dams (blank circles) in the Indo-Burma Region [13] with Mekong River shown in blue line. (b) Spatial arrangement of drainage basins, prior to construction of dams (i.e. ‘Pre-dam’ condition). (c) Fragmentation of the drainage basins due to man-made barriers, assuming that all planned dams are constructed. Note that the graphical images are illustrative only; see [13] for a precise map of Indo-Burma.

The Indo-Burma region has a dense and growing human population, and the twin imperatives of economic development and livelihood improvement have led governments to prioritize economic growth over environmental protection [16–17]. Such growth requires energy, spurring plans for construction of numerous hydropower dams globally and especially in Asia [13, 18–19] (Fig 1A). Hydropower dams offer an alternative to burning fossil-fuels for energy generation, and could contribute to reductions in CO_2_ emissions and a slower rate of global warming [20–21]. However, dams limit connectivity along river channels, disaggregating entire drainages (Fig 1B) into fragments (Fig 1C) [22], isolating fish populations and blocking their migrations [23]. Dams also disrupt downstream flood cycles, limiting the extent of floodplain inundation and thereby reducing fish production [15]. Longitudinal transport of sediment, nutrients and carbon are also affected [24] with downstream consequences such as delta shrinkage and saline intrusion that will be worsened as sea levels rise [25].

Global warming, which is generally attributed to rising CO_2_ emissions [26], represents a further threat to fish and fisheries in Indo-Burma, acting through temperature rises and changes in river flow [27–28]. Species can respond to such changes by shifting their climatic niche along three non-exclusive axes: time (e.g. phenology), space (e.g. range) and self (e.g. physiology) [29]; here we focus on the second of these. Fishes could, conceivably, adjust to rising water temperatures by making compensatory movements upstream to higher elevations or northwards where temperatures are cooler [8]. The presence of dams or other in-stream barriers prevent such movement so that range adjustment fails to track global warming [10, 30], and the potential for adaptation to warmer conditions is limited because tropical ectotherms are already close to their upper tolerance limits [31]. Although there is a paucity of research on the potential impacts of global warming on freshwater biodiversity in the tropics [8], including Indo-Burma [28, 32], we can be virtually certain that the effects of global warming and on-going dam construction in the region will combine to have synergistic effects on freshwater fishes.

The present study, using graphical species distribution models across large scales [33], is the first to present potential futures for fish biodiversity within the Indo-Burma hotspot under a range of scenarios arising from the individual and combined effects dam construction and global warming. We predict that hydropower dams will have greater and more immediate impacts than global warming on fish biodiversity in the Indo-Burma hotspot, especially within the LMB. Limiting dam construction–particularly on river mainstreams–should be a priority for conserving fish biodiversity and sustaining fisheries in the region.

## Materials and Methods

### Ethics statement

All the fish distribution data in the current study have been archived in a publically accessible online database [34] (http://ffish.asia).

### Fish distribution data

**Fig 2:**
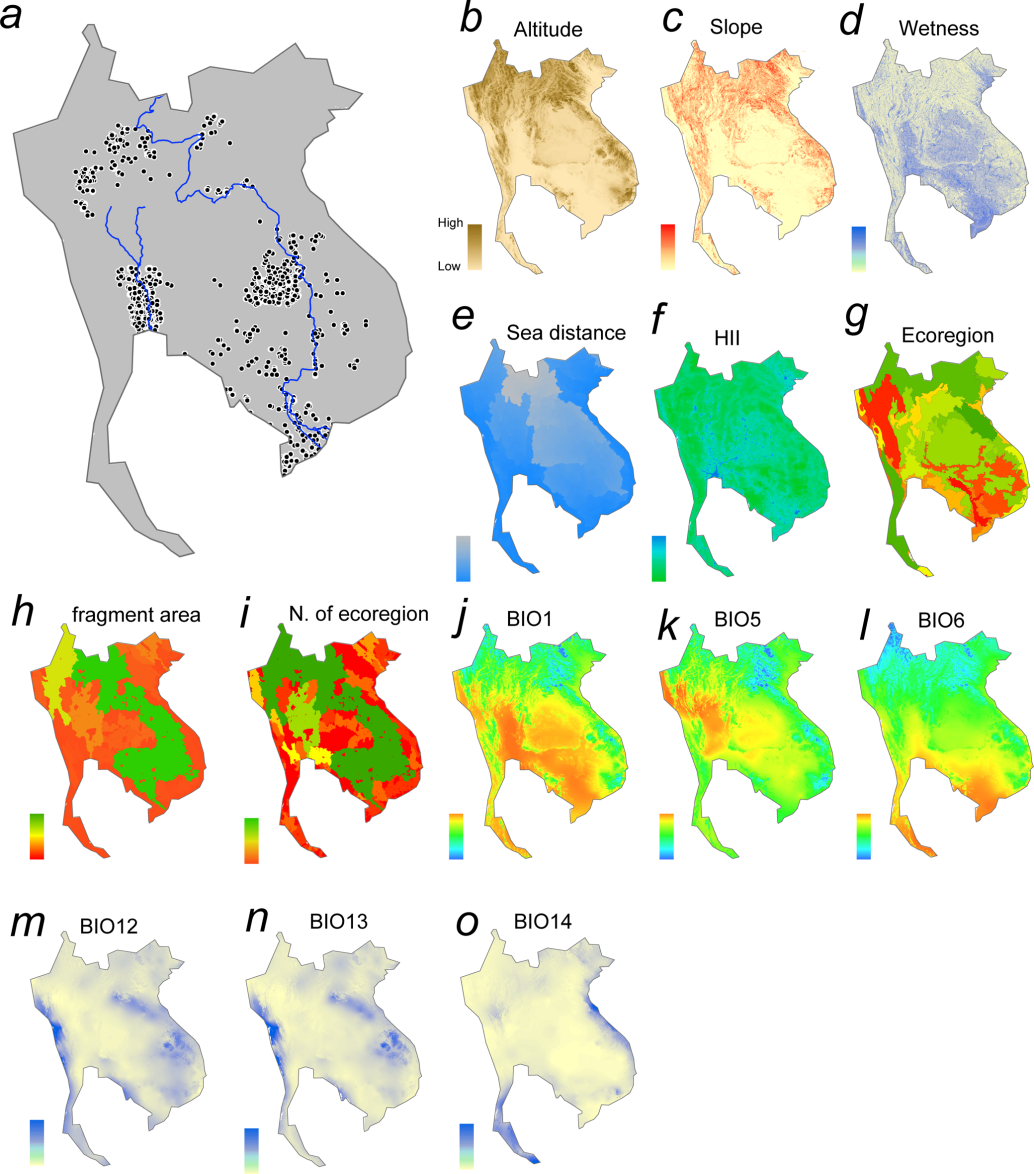
Sampling locations and environmental layers for MAXENT. (a) Sampling locations (solid circles) in the Indo-Burma region with the Chao Phraya (left) and Mekong (right). (b) Altitude layer obtained from USGS GTOP30 [41]. (c) Slope layer obtained from USGS HYDRO1K [42]. (d) Topographic wetness index [44] obtained from USGS HYDRO1K [42]. (e) Distance from the river mouth (or sea) derived from the altitude layer using GIS software. (f) Human influence index (v2, 1995–2004) obtained from SEDAC EARTH DATA [46]. (g) Ecoregions [47] obtained from WWF Terrestrial Ecoregions of the World [48]. (h) Fragment areas derived from the altitude layer and dam locations (Fig 1A) using GIS software. (i) Number of ecoregions within each fragment derived by intersecting the fragment layer and ecoregions using GIS software. (j) Mean temperature obtained from WorldClim global climat4 data (BIO1) [49]. (k) Maximum temperature obtained from the Bioclim data (BIO5) [49]. (l) Minimum temperature obtained from the Bioclim data (BIO6) [49]. (m) Precipitation obtained from the Bioclim data (BIO12) [49]. (n) Maximum precipitation obtained from the Bioclim data (BIO13) [49]. (o) Minimum precipitation obtained from the Bioclim data (BIO14) [49]. Color gradations show relative values in each layer. Note that the graphical images are illustrative only; readers are referred to the original sources of the environmental layers.

Fish distribution data were derived from an online database [34] that integrated information on freshwater fish specimens in collections around Southeast Asia. It comprised collections from 1571 sites in Cambodia, Laos, Thailand, and Vietnam (Fig 2A) made between 2007 and 2014 in the context of a large-scale project initiated by the Nagao Natural Environment Foundation, Japan [34–35]. The sampling sites comprised a variety of habitats including river mainstreams and tributaries, lakes and ponds, as well as swamps, marshes and ditches, at a wide range of elevations. In each location, fishes were collected with a variety of gear including cast nets, large and small seines, hand nets, hook-and-line, trawls and set-nets, for 1 to 4 hours along 50–100 m of shoreline. Due to the range of locations, habitat types, sampling gear and collectors involved, it was not possible ensure equality of sampling effort at each site. However, sampling continued until no new species were encountered at each location, and thus we hoped that collections included adequate representation of the dominant and common species at a site [35]. A total of 581 species were recorded in the database, but only data on 365 native species–each present in five or more sites–were included in our analysis. Species found at less than five sites were excluded to reduce the incidence of false negatives arising from insufficient sampling effort.

### Dam information

Locations of existing and planned dams within the Indo-Burma Region (Fig 1A) were manually plotted on GIS software (ArcGIS 10.2; ESRI Inc., USA) based on location information from literature sources [13, 36–37] and websites [38–39], with the positions existing dams checked from aerial photos using Google Earth. Totally, 596 existing dams were confirmed, at least 43 of them constructed between 1964 and 2003; six of those dams had reported generating capacities between 136 and 1500 MW, but such data from the others are lacking. A further 121 dams were planned or under construction, 25 of which had capacities between 6 and 3300 MW. Using data from the 31 dams of known generating capacity, the potential generating capacity of all other dams was estimated using a simple logarithmic regression model. It incorporated drainage-basin area above the dam and slope at the dam site (adjusted *R*^2^ = 0.51), assuming that greater flow (*P* < 0.0001) and steeper topography (*P* = 0.45) resulted in higher generating capacity. Based on this model, we derived generating capacities for all 717 (596 + 121) dams: they ranged from 1 to 3300 MW.

### MAXENT analysis for each fish species

We used MAXENT [40] to conduct species habitat suitability modelling under a variety of dam construction and global warming scenarios. MAXENT employs a maximum entropy modelling approach using inputs of environmental variables such as elevation, slope, temperature, etc. and species occurrence data to create a predictive model of habitat suitability for a given species. Once a model describing conditions suitable for species presence is built from co-occurring species presence and environmental data, MAXENT can use a combination of constant and changed environmental conditions to predict species occurrence probabilities under a variety of future scenarios.

MAXENT version 3.3.3k was applied to occurrence data for each of the 365 fish species in the Indo-Burma Region that were included within our database. We trained species occurrence models using 14 layers of environmental factors anticipated to have a direct or indirect influence on fishes (Fig 2B–2O). We then substituted them with layers that reflected changed conditions (e.g. higher annual mean temperatures) under different scenarios of dam construction and global warming in order to predict how species distributions would shift in response to future conditions. Of the 14 layers, altitude [41] and slope are basic topographic factors correlated with other environmental variables such as water temperature, water velocity, substrates size of stream beds, and discharge volume. The topographic wetness index is known to be accurate and correlated with floodplain extent [42–44], which constitutes important habitat for fishes [13], while distance from the sea was included to captures aspects of fish longitudinal zonation along rivers, especially the distribution of brackish species correlated with salinity [45]. The Global Human Influence Index v2 [46] was used as a composite measure of human impact. Ecoregional identity [47–48] was included to discriminate geographical assemblages of fishes. These six layers (Fig 2B–2G) were fixed in all projections made under different scenarios in MAXENT analysis. In contrast, layers related to dam construction–i.e. fragment areas (i.e. the extent of individual fragments within drainages upstream of each dam), and the number of ecoregions within each fragment [22]–changed under different dam and global warming projections (for details, see below): the cell values of these two layers decreased as the number of dams increased and fragmentation became greater.

For global warming, we used six climate layers derived from WorldClim global climate datasets [49]: annual mean temperature, maximum temperature of the warmest month, minimum temperature of the coldest month, annual precipitation, precipitation during the wettest month, and precipitation during the driest month. Although WorldClim comprises a total of 19 bioclimatic layers (BIO_1_–BIO_19_ for current, 2050 and 2070), but we selected only six of them to reduce redundancy due to correlation among variables; trial analyses revealed their inclusion did not increase analytical precision.

All 14 environmental layers encompassed the entire Indo-Burma Region, including Thailand, Laos, Cambodia, Vietnam and eastern Myanmar [13] (Figs 1 and 2), and were constructed using the ArcGIS with a resolution of 30 arc seconds square (approximately 1 km^2^) as used by WorldClim. We ran MAXENT for each species with default settings as follows: random test percentage = 25; regularization multiplier = 1; maximum number of background points = 10,000. To address sampling bias [50–52], we created a sampling bias file according to the collecting effort: e.g. if two sampling locations were included in a 30-sec square cell, a value of 2 was given for that cell. The MAXENT results yielded a layer of habitat suitability (0–1) for each fish species in each cell (S1 Fig). All but two of the 365 species analyzed had an AUC (area under the curve) statistic >0.7, and these 363 species were subject to further analysis (S1 Table; S1 File). For the projections of the effects of different scenarios of dam construction and global warming, current species occurrence model was trained using the 14 current layers for each species, and the ‘Projection layers directory/file’ function of MAXENT was applied to the 14 scenario-layers to project changes in species occurrence in future.

As we were only interested in the overall model accuracy, and not in the interpretation of variables that contributed to the model, we did not undertake any model selection but used all the 14 layers at once; other research has shown that this approach makes no essential difference to projected outcomes [52–53].

### Biodiversity responses

Three aggregate indicators of the effects of dams and global warming on fishes were developed to reveal responses in terms of changes in local species richness, habitable area, and the proportion of threatened species. First, a graphical map of local ‘species richness index’ was created by summing all 363 species layers from MAXENT, whereupon species richness index was calculated as the overall habitat suitability [54] of all species in each cell of the map. The mean of the species richness index was calculated by averaging values for all the cells of within the area of interest, specifically Indo-Burma as a whole but also within the LMB and within each country.

Second, we determined an average ‘habitable area index’ for each of the 363 species, with the area occupied by a species calculated from those cells that had a habitat suitability of at least 0.05 (S1 Fig). As there is no method for deriving species distributions from values of habitat suitability, the use of a threshold of 0.05, although perhaps somewhat arbitrary, follows the general custom of α = 0.05 in statistical testing. The mean of the habitable area index was also calculated by averaging this index across all 363 species.

The third index was the ‘proportion of threatened species’, defined as species predicted to decline by over 30% in habitable area index compared to ‘pre-dam’ conditions. The IUCN Red List of threatened species [55] treats any species that has experienced such a decline as ‘vulnerable’ (and, in more extreme cases, as ‘endangered’ or even ‘critically endangered’), but for the purposes of the present study, our projections merely distinguished between those species that became threatened (>30% decline) and or remained non-threatened (<30% decline). ‘Pre-dam’ as used here corresponds to a scenario in which all existing dams within the Indo-Burma region were assumed to have been removed (see below, 100% dam-removal scenario).

### Dam scenarios

We developed four algorithms to derive to possible scenarios for dams and electricity-generating capacity. First, for each planned dam, regardless of location (mainstream or tributaries), we assumed a probability of construction ranging from 5% to 100% increasing at increments of 5%, giving rise to a total of 20 ‘Planned dam’ scenarios (S2 Table; Scenario IDs: 1–20). Second, for each planned dam on the mainstream of the Lower Mekong (i.e. downstream of the border of China), we again assumed a probability of construction ranging from 5% to 100%, with 5% increments, resulting in 20 ‘Mainstream dam’ scenarios (S2 Table; Scenario IDs: 21–40). Third, another 20 ‘Tributary dam’ scenarios for the construction of dams on Mekong tributaries and other rivers outside the Mekong drainage using the same range of probabilities (S2 Table; Scenario IDs: 41–60). Fourth, existing dams, regardless of location, were assumed to have a probability of removal ranging from 5% to 100%, with 5% increments, yielding 20 dam-removal scenarios (S2 Table; Scenario IDs: 61–80). We used the ‘RAND’ function of Microsoft Excel to randomly allocate the possibility of construction or removal to individual dams under each scenario, with each dam allocated a value between 0–1; if the value was lower than the possibility that a dam would be constructed, then it was assumed the dam would have been built (e.g., a dam with value 0.031 in the ‘5% planned dam’ scenario would have been built, but one with a value of 0.072 would not). Then, taking account of the dams that have already been constructed within the region (S2 Table; Scenario ID: 0), MAXENT was used to analyze a total of 81 dam-construction and electricity-generating scenarios for the 363 fish species under present-day climatic conditions.

### Global-warming scenarios

We derived future climate scenarios from WorldClim CMIP5 global climatic data where 19 models predict climate under four representative concentration pathways (RCPs) of +2.6, +4.5, +6.0, and +8.5 W/m^2^ relative to pre-industrial values [26, 56] for 2050 and 2070 (note: several of these models lack +2.6, +6.0, and/or +8.5 RCPs). A total of 126 scenarios were then analyzed using MAXENT to yield projections for the 363 fish species, taking into account those dams that have already been constructed within the region; i.e. the ‘current dam’ condition (S2 Table; Scenario IDs: 81–206). As with layers related to dam construction, the cell values for these layers were not held constant during projections from global warming scenarios, but changed according to the climate projection used.

### Additive scenarios

We developed additive scenarios that could be used to make projections by randomly allocating an arbitrary subset of 81 of the 126 global-warming scenarios to the 81 dam-construction (and, hence electricity-generating) scenarios. The scenarios were ordered by the value generated by the Excel RAND function (see above) within respective global-warming and dam-construction scenarios, and scenarios with the same rank were paired as a subset. The 81 additive scenarios were then analyzed using MAXENT to yield projections for the 363 fish species (S2 Table; Scenario IDs: 207–287).

### Synergistic scenarios and Δ

Here we assumed that compensatory range shifts of fishes in response to warming was inhibited by dams acting as barriers and preventing them from moving to parts of the river network that would otherwise offer suitable thermal habitat. To make the consequent projections, we assumed that once a species had been lost from a particular fragment under a particular dam-building scenario, it would not reappear within that fragment even if thermal conditions (under one of the global-warming scenarios) become suitable. We treated a species as absent from fragment if it contained no cells with a habitat suitability >0.05 (S2 Fig). This algorithm was applied to the 81 additive scenarios, to yield a further 81 synergistic scenarios (S2 Table; Scenario IDs: 288–368). We then calculated the difference, i.e. Δ, between the 81 projections arising from the additive and synergistic scenarios for each of the three biodiversity responses.

### Overall trends of projections

We undertook generalized additive modelling (GAM) to compare general trend and predictive accuracy the dam scenarios and global-warming scenarios. For projections based on the 81 dam scenarios, GAM was conducted for each of the three biodiversity response indices (as dependent variables) versus the combined electricity-generating capacity of all dams (independent variable). For projections based on the 126 projection scenarios. GAM was conducted for each biodiversity response index *versus* RCP (independent variables) for each year (2050 and 2070).

For both the 81 synergistic scenarios and the difference (Δ) between the additive and synergistic scenarios (i.e. the portion attributable to synergy), we conducted stepwise multiple regression in both directions for each biodiversity response index *versus* the combined electricity generating capacity of all dams, RCPs and year (independent variables). Regression analyses were calculated using R (version 3.13) (http://www.r-project.org), with the functions ‘lm’ and ‘stepAIC’ deployed regression and variable selection, respectively. For improve normality and correct homoscedasticity, all the variables were standardized with the ‘Standardize’ function of Microsoft Excel.

## Results

### Projections of overall impacts upon fishes

**Fig 3:**
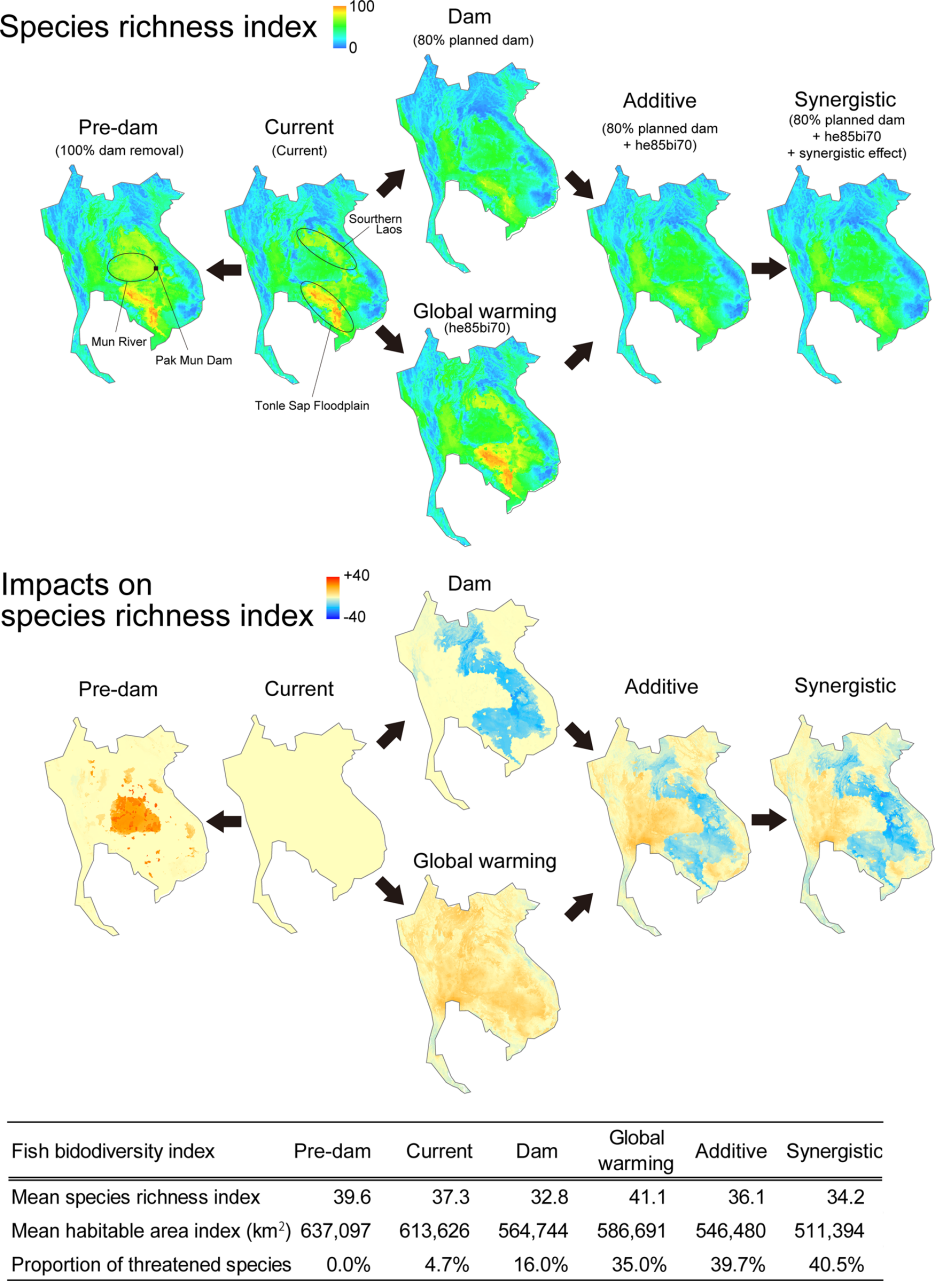
A sequence of changing fish biodiversity under six representative scenarios of dam construction/removal, global warming, and the simple addition or synergy between these two threat factors. Scenario names in parentheses correspond to those in S2 Table.

Here we highlight six representative projections (Fig 3) among the 369 scenarios generated (S2 Table). The present-day pattern of fish biodiversity (Current: Fig 3) showed that species richness index was especially high in the Tonle Sap floodplain and in southern Laos, but that around 5% of the 363 species included in our analysis were categorized as threatened. The prevailing intra-regional differences in species richness index (min–max: 7.2–94.7) were reduced under the dam-construction projection (min–max: 7.2–78.3), although Tonle Sap remained the most species-rich area (Dam: Fig 3). Overall, this dam-only projection yielded the lowest mean species richness index, with 16% of all species categorized as threatened. If all the dams in the region were removed (Pre-dam: Fig 3), there was an increase (roughly +2) in mean species richness index; a localized recovery along the Mun River in Thailand, fragmented by the Pak Mun Dam, was notable also. A global-warming projection under the ‘he85bi70’ climate model (Global warming: Fig 3) yielded an increase in mean species richness index throughout the study area (min–max: 7.2–94.5 per cell) to a level greater than that seen in the pre-dam projection, although the proportion of threatened species rose to over one third of all fishes–twice that under the dam-only projection.

When the dam and global-warming scenarios were simply added together (Additive: Fig 3), species richness index declined to a little below that currently prevailing across the region (min–max: 6.4–77.1), but was higher than under the dam-only projection. However, a marked decline in mean habitable area index increased the proportion of threatened species to 41.1%. In scenarios which assumed that the presence of dams would inhibit compensatory range shifts by fishes (Synergistic: Fig 3), mean habitable area index decreased even further, and was the lowest of any scenario shown in Fig 3. The species richness index (min–max: 6.0–77.0 per map cell) was higher than under the dam-only and additive projections, while the proportion of threatened species increased to 40.5%.

### Dam impacts

**Fig 4:**
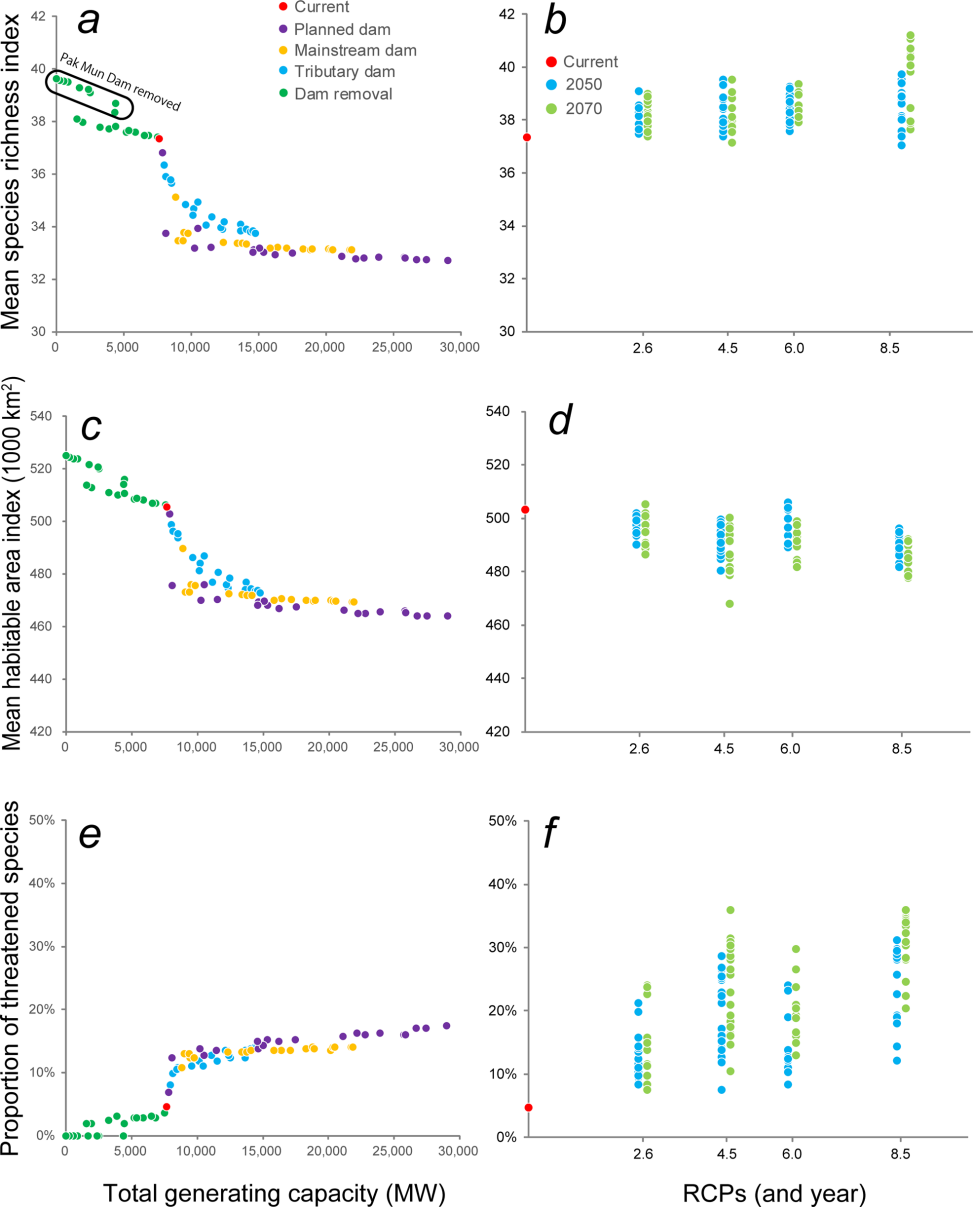
Effects of dam construction (represented by generating capacity) and global warming (reflected in RCPs and year) on fish biodiversity. Changes in mean species richness projected to arise from (a) increases in total generating capacity (associated with hydropower dams), and (b) RCPs and year (associated with global warming). Changes in mean habitable area projected to arise from (c) increases in total generating capacity, and (d) RCPs and year. Changes in % threatened species projected to arise from (e) increases in total generating capacity, and (f) RCPs and year.

Disparities among projections from different dam-building scenarios were attributable mainly to variations in total electricity-generating capacity (Fig 4A, 4C and 4E). The GAM analysis (S3 Fig) showed that mean species richness index (*P* < 0.0001; adjusted *R*^2^ = 0.94; deviance explained = 94.9%, GCV = 0.32) and mean habitable area index (*P* < 0.0001; adjusted *R*^2^ = 0.95; deviance explained = 95.2%, GCV = 23.2) declined as total generating capacity increased, while the proportion of threatened species (*P* < 0.0001; adjusted *R*^2^ = 0.96; deviance explained = 96.5%, GCV = 1.35) showed the opposite trend. In all three models, the decline/increase in the biodiversity indices were most apparent over the 7600 MW to 15,000 MW range.

Projections from ‘Planned dams’ scenarios, which assumed that various proportions of planned dams were constructed without regard to whether they were on mainstream or tributaries, generally resulted in the most conspicuous impacts on fish biodiversity, but projections under from the Mekong ‘Mainstream dams’ scenarios were only slightly smaller. Projections based on scenarios that involved construction of various proportions of planned ‘Tributary dams’, without any Mekong mainstream dams, considerably smaller impacts on biodiversity. However, their magnitude was sensitive to combined generating capacity, especially in the 7600 MW to 10,000 MW range where the proportion of threatened species rose from 3% to 12% (Fig 4E). Projections from a set of dam-removal scenarios, intended to indicate the ‘pre-dam’ baseline for fish biodiversity in the absence of existing dams, were associated with higher values of the mean species richness index and mean habitable area index, as well as a reduction in the proportion of threatened species to near zero. The biggest recovery of species richness was seen in projections that included removal of Pak Mun Dam (Fig 4A, see also Fig 3).

**Fig 5:**
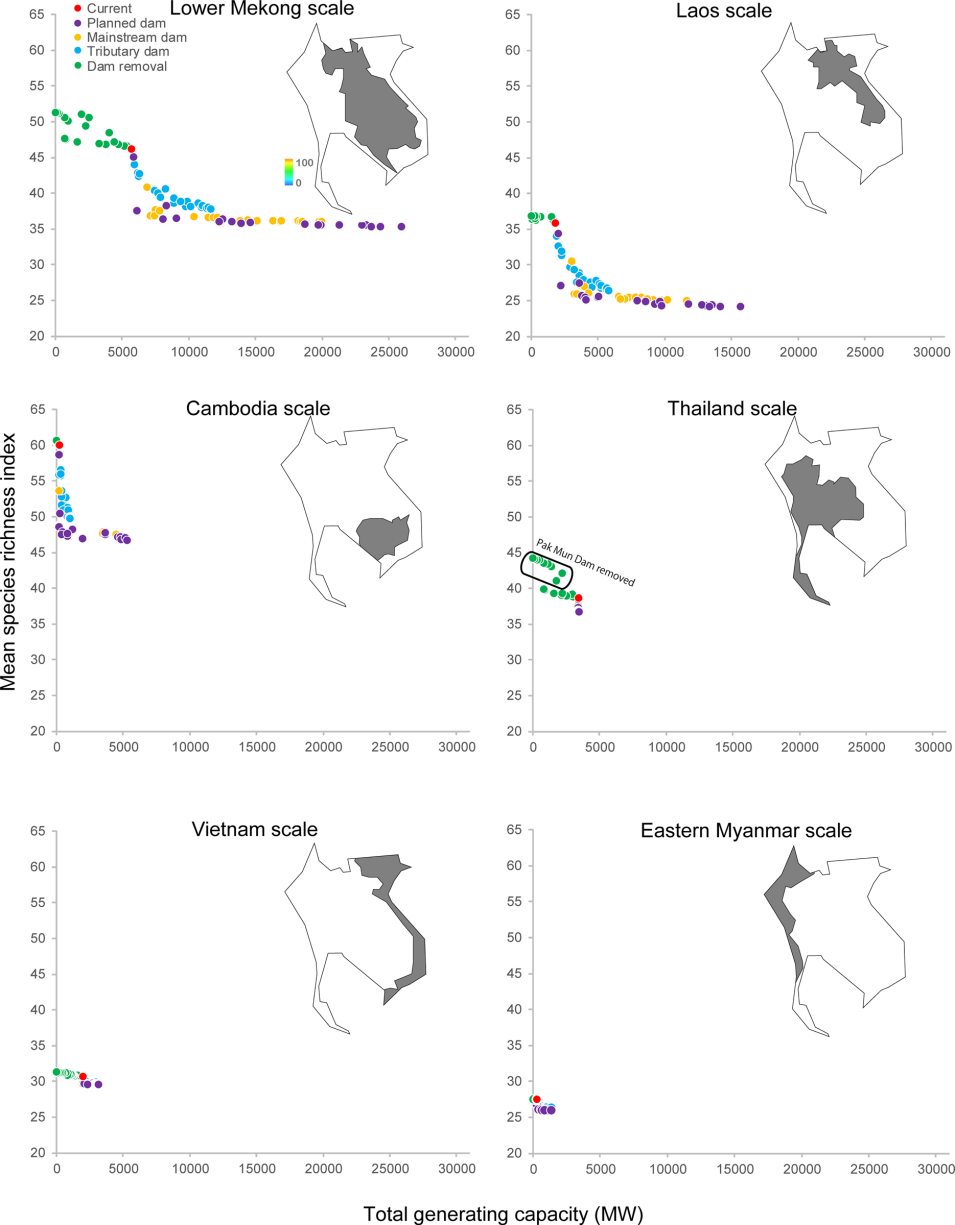
Changes in fish biodiversity dues to dam construction at the intraregional scale within the Indo-Burma Region.

Responses of biodiversity to dam construction differed spatially within the Indo-Burma Hotspot (Fig 5), with greatest declines in the mean species richness index (by 31–32%) within the LMB generally–especially within Laos (34–35%). In addition, substantial reductions in the mean species richness index (22–23%) were projected for Cambodia. Although the increase in hydropower capacity within Cambodia is projected to be relatively modest under all scenarios, river fishes will be affected by dams in Laos immediately upstream of the Cambodian national boundary.

### Global-warming impacts

The three biodiversity indices showed changes under global warming (Fig 4B, 4D and 4F), but the responses were less clear-cut than under the dam-building scenarios. GAM analysis (S4 Fig) of the response of the mean species richness index (Fig 4B) to increasing RCPs was unclear in 2050 (*P* > 0.05; adjusted *R*^2^ = 0.04), but was comparatively more distinct in 2070 when richness tended to show small increases under higher RCPs (*P* < 0.0001; adjusted *R*^2^ = 0.39). In contrast, the mean habitable area index tended to decline under higher RCPs (Fig 4D) in both 2050 (*P* < 0.0001; adjusted *R*^2^ = 0.32) and, especially in 2070 (*P* < 0.0001; adjusted *R*^2^ = 0.28). The proportion of threatened species tended to rise as RCPs increased (Fig 4F) in 2050 (*P* < 0.0001; adjusted *R*^2^ = 0.33) and, as with habitable area, the response was clearer in 2070 projections (*P* < 0.0001; adjusted *R*^2^ = 0.48). Importantly, some global-warming projections gave rise to a higher proportion of threatened species (up to 36%; Fig 4F) than arose from dam impacts (17%; Fig 4E) because changes in the habitable area index under some climate projections were more substantial than those attributable to dams (S5 Fig) and exceed the 30% threshold that was indicative of threatened species.

### Synergistic impacts and Δ

**Fig 6:**
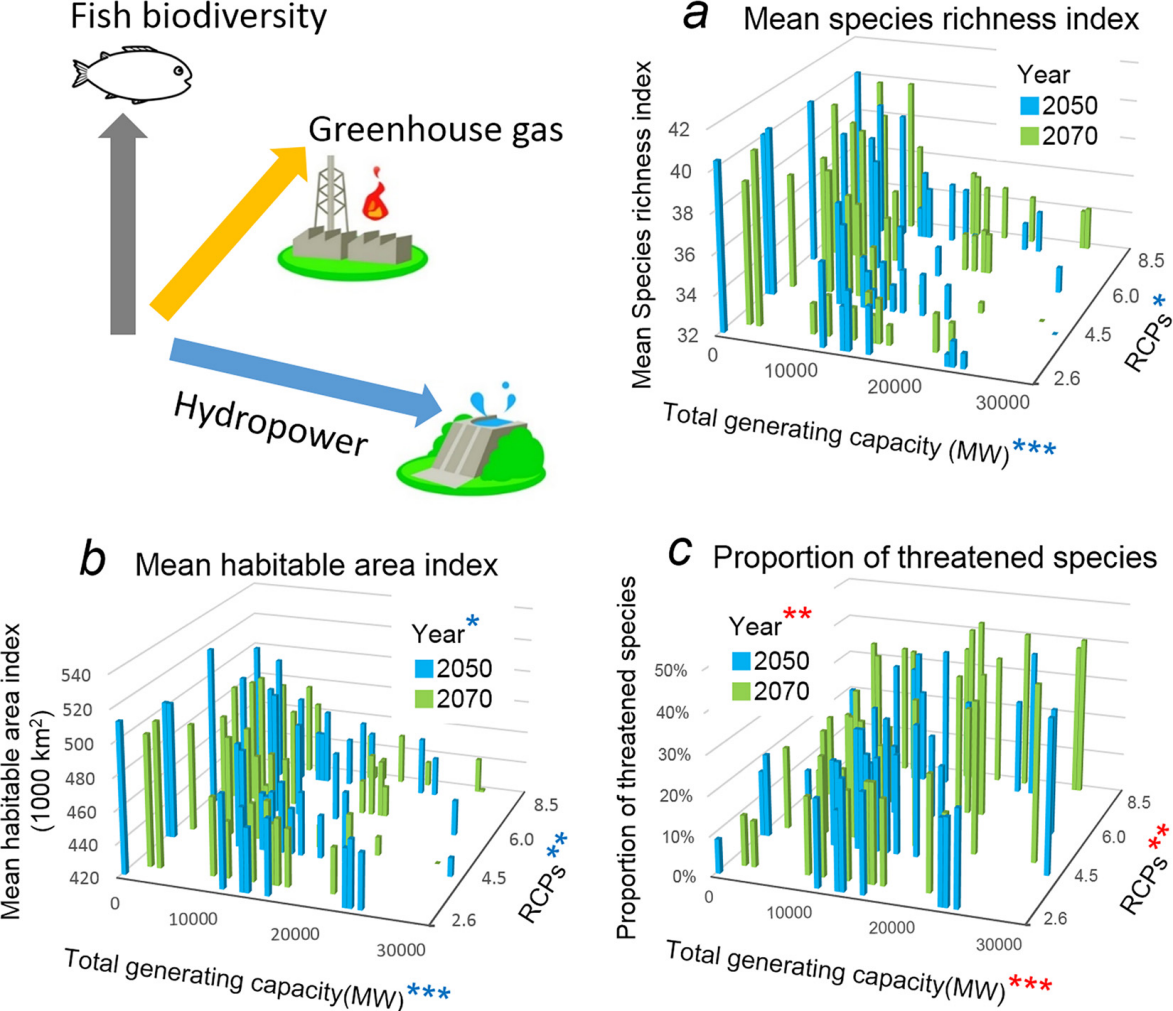
Synergistic impacts of dam construction and global warming on fish biodiversity. Changes in (a) species richness, (b) habitable area, and (c) % threatened species projected to arise from increases in total generating capacity (associated with hydropower dams), RCPs and year (associated with global warming). *P < 0.05, **P < 0.01, ***P < 0.001. Red and blue asterisks indicate positive and negative effects, respectively.

The mean species richness index under synergistic scenarios (Fig 6A, adjusted *R*^2^ = 0.69) was negatively correlated with total generating capacity (*P* < 0.001) and positively correlated with higher RCPs (*P* < 0.05), but not sensitive to year; the magnitude of reductions in mean species richness index (Fig 6A) were of a similar to those arising from dams alone (Fig 4A). The mean habitable area index in the synergistic scenarios (Fig 6B, adjusted *R*^2^ = 0.71) was negatively correlated with total generating capacity (*P* < 0.001), RCPs (*P* < 0.01) and year (*P* < 0.05), and showed a greater decrease than observed under either the dam-building or global-warming projections (Fig 4C and 4D), most notably in projections of synergistic scenarios with high generating capacity. Likewise the proportion of threatened species under synergistic scenarios (Fig 6C, adjusted *R*^2^ = 0.54) was negatively correlated with total generating capacity (*P* < 0.001), RCPs (*P* < 0.001) and year (*P* < 0.01), with values (7–44%) also exceeding those associated with dam construction or global warming alone (Fig 4E and 4F; range: 0–36%).

**Fig 7:**
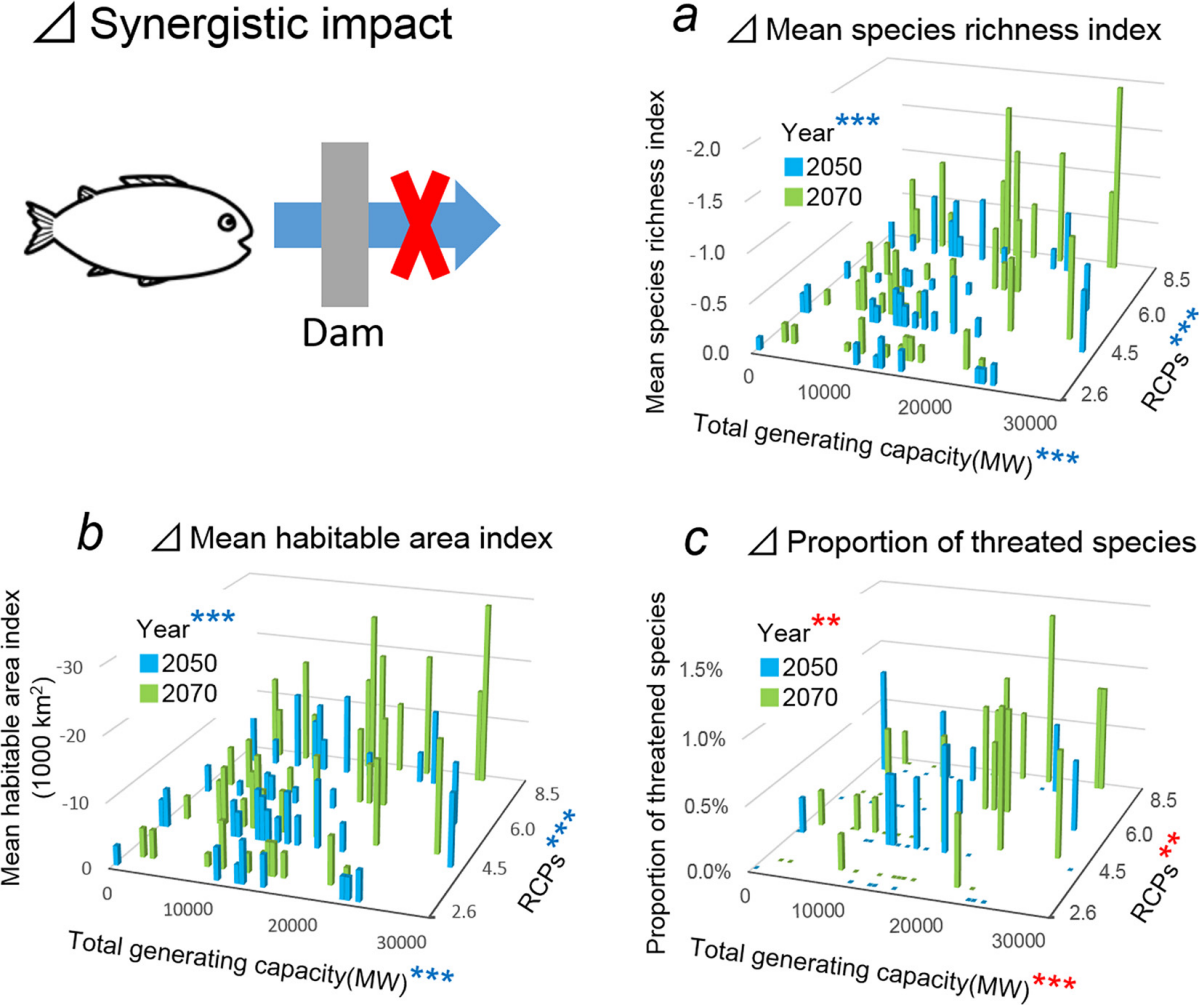
The proportion of impacts on fish biodiversity from dam construction and global warming that is attributable to synergy alone (i.e. the difference, Δ, in projections between the additive and synergistic scenarios). (a) Δ species richness, (b) Δ habitable area, and (c) Δ % threatened species projected to arise from increases in total generating capacity (associated with hydropower dams), RCPs and year (associated with global warming). **P < 0.01, ***P < 0.001. Red and blue asterisks indicate positive and negative effects, respectively.

The differences, Δ, between the additive and synergistic projections for the mean species richness index (Fig 7A, adjusted *R*^2^ = 0.48) and mean habitable area index (Fig 7B, adjusted *R*^2^ = 0.50) were negatively correlated with total generating capacity (*P* < 0.001), RCPs (*P* < 0.001) and year (*P* < 0.001). The Δ in terms of % threatened species (Fig 7B, adjusted *R*^2^ = 0.28) was likewise positively correlated with total generating capacity (*P* < 0.001), RCPs (*P* < 0.01) and year (*P* < 0.01). While these findings clearly indicate the synergistic impacts of global warming and dam construction on all three biodiversity indices, the on threatened species were less than those on species richness and mean habitable area: with a single exception (Fig 7C), Δ values were <1%

## Discussion

Our analyses indicate that dams will have a significant impact on fish biodiversity in the Indo-Burma hotspot and particularly within the LMB. The extent and intensity of impact will depend on both the location of the dams (with mainstream dams being particularly damaging) and their combined generating capacity. Especially marked responses in biodiversity indices are predicted as capacity increases from the present level (7600 MW) up to around 15,000 MW. In part this is because dams are planned in locations where their downstream impacts would influence the Tonle Sap floodplain and southern Laos, areas of the highest species richness index. Indeed, at the time of writing, on-going construction work on the Xayaburi Dam in Laos, and site formation for a second dam at Don Sahong, just upstream of the border with Cambodia, are clear and present threats to the Mekong fishes and the capture fishery [13, 22].

Our results verify previous predictions that construction of mainstream dams would be more detrimental to LMB fisheries than that of tributary dams [19, 22]. We show that tributary dams will have smaller impacts on fish biodiversity, so long as their combined generating capacity does not exceed 15,000 MW, whereas any scenario involving mainstream dams would have substantial impacts on all three indices of fish biodiversity. Nonetheless, since tributaries are important passages for many fishes undertaking breeding migrations within the LMB [57], all dam-construction scenarios result in some trade-off of between fish biodiversity and hydropower generation [23]. We also show that removal of existing dams such as Pak Mun, where the fishery collapsed after a dam was completed in 1994, would have positive effects on fish biodiversity, and this accords with local increases fish catches reported after a one-year trial opening of the dam gates [58]. Although such dam removal would have positive effects, the avoidance of construction of further dams is a much higher conservation priority for the LMB than removing dams that are already present (even if that were possible).

The projected effects of global warming appeared to be generally smaller than those attributable to dam construction, but nevertheless resulted in a marked increase in the proportion of species categorized as threatened regardless of the extent to which local species richness was affected. In fact, the mean species richness index increased under some scenarios, and the effects of global warming on fish biodiversity varied considerably among RCPs, and the extent to which individual species were impacted or favored. By contrast, dams had more similar (generally negative) effects on most species. While dam construction and global warming had distinct and independent effects on fish biodiversity, their synergistic effects were considerably more marked than their simple additive effects. Perhaps inevitably, the synergistic impacts became more noticeable as generating capacity increased, most obviously under scenarios involving higher RCPs, and became larger over time (from 2050 to 2070).

Our projections may have some limitations. The bioclimatic envelope model (i.e. MAXENT) that we used has the advantage that it can be applied to a large number of species or taxa, and can capture many ecological processes inherent the relationship between occurrence data and spatial information. However, a disadvantage is that it does account for the mechanisms that mediate species ranges, and contains the assumption that the current distribution of a species is a reliable indicator of suitable climate [29]. In addition, MAXENT may handle novel climates poorly and, given the uncertainty inherent in any predictions of range shifts in response to global warming [29], there is inevitable imprecision in the absolute magnitude of global warming impacts, as well as the additive/synergistic impacts of dams and climatic warming on fish biodiversity. Although estimates of the absolute magnitude of projected impacts under different scenarios of dams and climate warming may be imprecise, and thus our estimates of the exact values of changes in the species richness index or reductions in habitable area may contain some inaccuracies, the relative magnitude of these impacts under different scenarios is likely to be more robust. In other words, we have more confidence in our prediction that the synergistic impacts of dams and warming on fishes will be more damaging than their additive effect, than we would be in making a precise estimate of (say) the reduction in mean species richness under a particular scenario.

In our analysis, some MAXENT species layers (S1 Fig) comprised only 5–10 distributional records (S1 Table). We believe, however, the large number of species in our analysis, and the range of families represented, could enhance the robustness of our predictions and compensate for errors in forecasts about changes in fish biodiversity relative to those based on smaller numbers of species or a more limited array of taxa [8]. Furthermore the patterns of freshwater fish species richness index in our graphical map of the Indo-Burma region (Fig 3) is quite similar to those at based on an independent dataset compiled for that region (see Figure 3.2 of Allen et al. [13]), even though our richness map with that derived independently by Allen et al. [13]. While MAXENT provides an index of relative habitat suitability and does not directly estimate occurrence probability [54], but our species richness index can be used to represent species richness in a particular map cell: for example, a total of 149 fish species have been recorded from Tonle Sap [59], and our prediction of 90–100 species richness index per ~1 km^2^ square around Tonle Sap under the six representative scenarios shown in Fig 3 seems well within the correct order of magnitude, when beta diversity across the entirety of the lake (15,000 km^2^) is considered.

We used a threshold value of 0.05 habitat suitability to determine the presence or absence of a species from a map cell. This value might be considered to be somewhat low for a determination of species absence, and thus habitable area might have been overestimated for some species. However, any threshold value used to define absence is in some sense arbitrary and we believe the general trends and relative responses of the biodiversity indices would be relatively insensitive to the threshold value used.

Our projections of the impacts of global warming (without dams) on fish species richness and mean habitable area are considerably less than that foreseen in some other studies [60–61], and are more congruent with a recent forecast of few species losses with no extinctions predicted (by 2090) for most drainage basins globally [62]. These authors note that non-climate related anthropogenic influences (e.g. dams) are far more likely to influence fish communities in the near future. Others suggest that colonizations and extirpations could play counterbalancing roles in reshuffling of fish communities, resulting substantial species turnover [63]. We note that the impacts of dams might be mitigated by fish passages or fish ladders, but only a few dams in Indo-Burma (e.g., at Pak Mun) have such structures. Furthermore their efficacy for Asian fishes is likely to be low [64], and such facilities to not compensate for the impacts on fishes other than those attributable to the physical barrier [65]. Accordingly we did not take account of any possible role of fish passages in predictions about the impacts of dams.

It may be difficult to extrapolate directly from the results of this study to the possible impacts of damming and global warming on fisheries in the Indo-Burma region in general and the LMB in particular. Many of species considered herein are not commercially important fishery species and thus our data on richness and habitable area cannot be directly related to predictions about changes in fishery yields (i.e., reductions in biomass). However, given the large number of species included in our study, some of which may be prey of fishery species, and the significant proportion of migratory species contributing to the Mekong fishery [14–15], our forecast of the strong and imminent impacts of dam construction within the LMB (especially on the Mekong mainstream) is surely a matter warranting attention. Replacement of the animal protein provided by the LMB fishery could require the equivalent of ~24,000 km^2^ of new pastureland for livestock [16]. In addition to such livelihood concerns, Mekong fishes have cultural significance for the region’s inhabitants [66].

Our data suggest that construction of dams on the lower Mekong mainstream would impact fish diversity and give rise to transboundary effects that would be felt in Cambodia beyond the immediate footprint of planned dams (Fig 5). However, some dam construction on tributaries may be possible without major impacts provided that the additional combined generating capacity does not exceed 1000–2000 MW (see, for example, Fig 4A). While dam construction will have far greater impacts on fish diversity than global warming, our projections show that the presence of dams will exacerbate the impacts of warming because they prevent fishes from making range adjustments in response to rising temperatures (see, for example, S2 Fig). Not only are the impacts of dams on fish biodiversity likely to be greater and to occur sooner than those attributable to global warming, we stress that they can be predicted with a higher degree of certainty.

At the time of writing, most of the Mekong remains free flowing and, thus far, construction of only one mainstream dam at Xayaburi appears inevitable. Whatever other dams are built is, ultimately, a decision that involves a wider constituency than scientists. Full engagement among all potential stakeholders will be an essential and immediate requirement for any attempts to conservation of fish biodiversity within the Indo-Burma hotspot [6]. Decisions made about the LMB, in particular, should be the subject of international consensus, involving all riparian states, and predicated on thorough environmental impact assessments that take due account of ecological, social and welfare issues within a wider economic framework [67]. While we do not expect that concerns about biodiversity conservation will necessarily trump decisions about dam construction and associated economic development in South East Asia, we hope that scientific projections such as this one will help inform such decisions, and thereby contribute to environmental sustainability.

## Supporting Information

S1 Fig Example of MAXENT results. Graphical maps show the habitat suitability for shovel-jaw carp, *Onychostoma gerlachi* (Cyprinidae), under projections derived from different scenarios. Scenario names in parentheses correspond to those in S2 Table. Solid lines in the maps show the distribution threshold (habitat suitability: 0.05) of *O*. *gerlachi*.
(TIF)

S2 Fig An example of synergistic effects. Compared to the dam-construction scenario, the distributional range of mudcarp, *Cirrhinus jullieni* (Cyprinidae), was projected to expand under the synergistic effects of dams and global warming. However, because of drainage-basin fragmentation caused by dams, the full expansion of distribution that would have occurred under global warming was prevented. Scenario names in parentheses correspond to those in S2 Table. Solid lines on the maps show the distribution threshold (habitat suitability: 0.05) of *C*. *jullieni*.
(TIF)

S3 Fig GAM analysis for total generating capacity versus three biodiversity indices. The blue line indicates the spline curve.
(TIF)

S4 Fig GAM analysis for different RCPs under global warming versus three biodiversity indices in 2050 and 2070. The blue line indicates the spline curve.
(TIF)

S5 Fig Differences in response patterns to dams and global warming. Most fish species showed a contraction in range extent under the planned-dam scenarios (assuming 100% of planned dams were built), while global-warming scenario ip85bi70 had little effect on average habitable area, although the extent of variation was sensitive to species identity. A similar general tendency was apparent in projections from other dam and global-warming scenarios. Solid circles indicate each species and a blue line indicates the threshold of increase/decrease. Species plotted in the orange sector are those that can be considered as threatened. Scenario names in parentheses correspond to those in S2 Table.
(TIF)

S1 File Fish distribution data. (CSV)

S1 Table Species list used in the analysis. (PDF)

S2 Table Scenario names and details. (PDF)
