# Supplementary figures and images for: Impacts of Dams and Global Warming on Fish Biodiversity in the Indo-Burma Hotspot
Source: PLoS One. 2016 Aug 17;11(8):e0160151. doi: 10.1371/journal.pone.0160151 (PMC4988766; doi:10.1371/journal.pone.0160151)

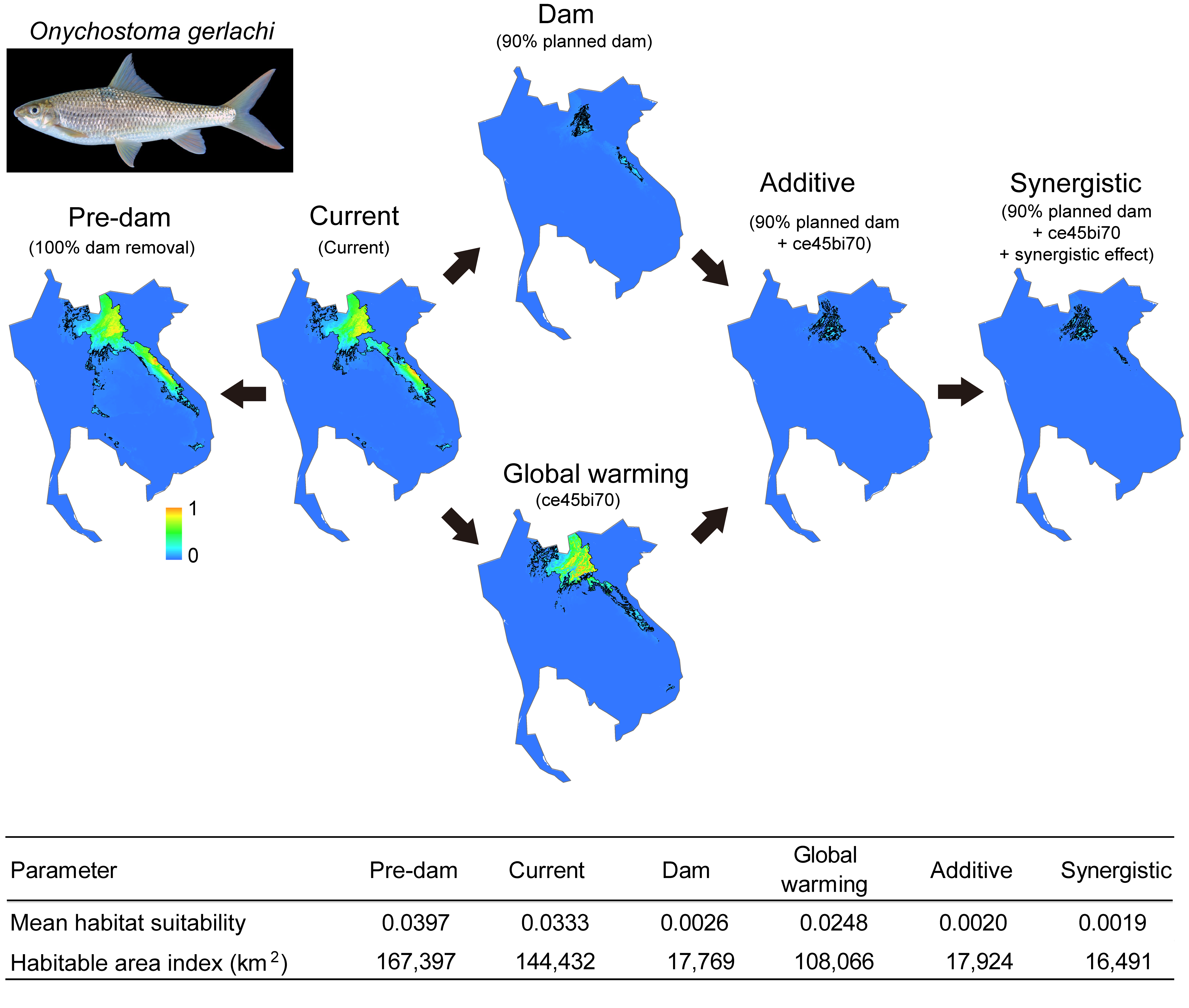

Supplement: S1 Fig — Graphical maps show the habitat suitability for shovel-jaw carp, Onychostoma gerlachi (Cyprinidae), under projections derived from different scenarios. Scenario names in parentheses correspond to those in S2 Table. Solid lines in the maps show the distribution threshold (habitat suitability: 0.05) of O. gerlachi. (TIF) [file pone.0160151.s001.tif]

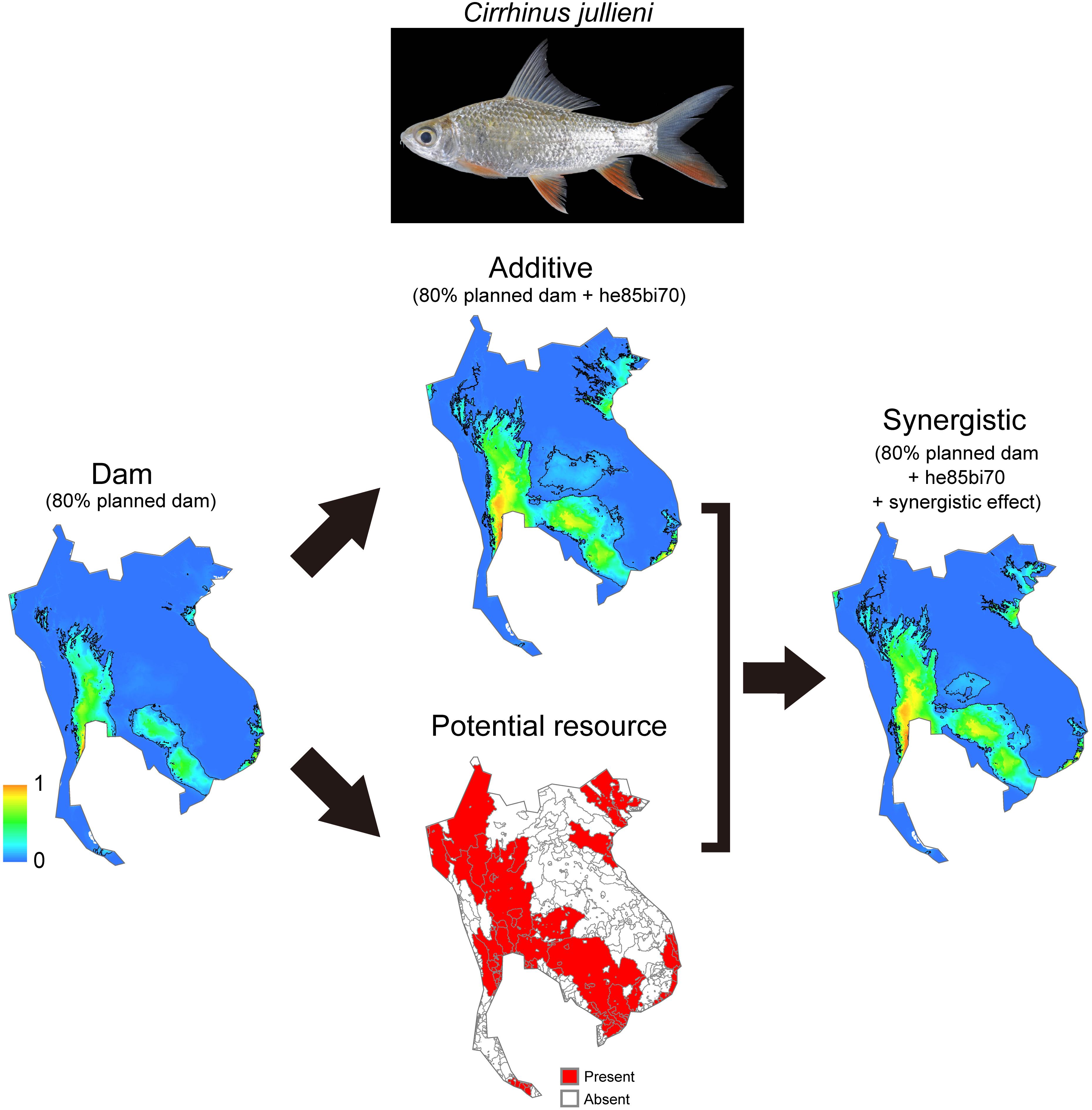

Supplement: S2 Fig — Compared to the dam-construction scenario, the distributional range of mudcarp, Cirrhinus jullieni (Cyprinidae), was projected to expand under the synergistic effects of dams and global warming. However, because of drainage-basin fragmentation caused by dams, the full expansion of distribution that would have occurred under global warming was prevented. Scenario names in parentheses correspond to those in S2 Table. Solid lines on the maps show the distribution threshold (habitat suitability: 0.05) of C. jullieni. (TIF) [file pone.0160151.s002.tif]

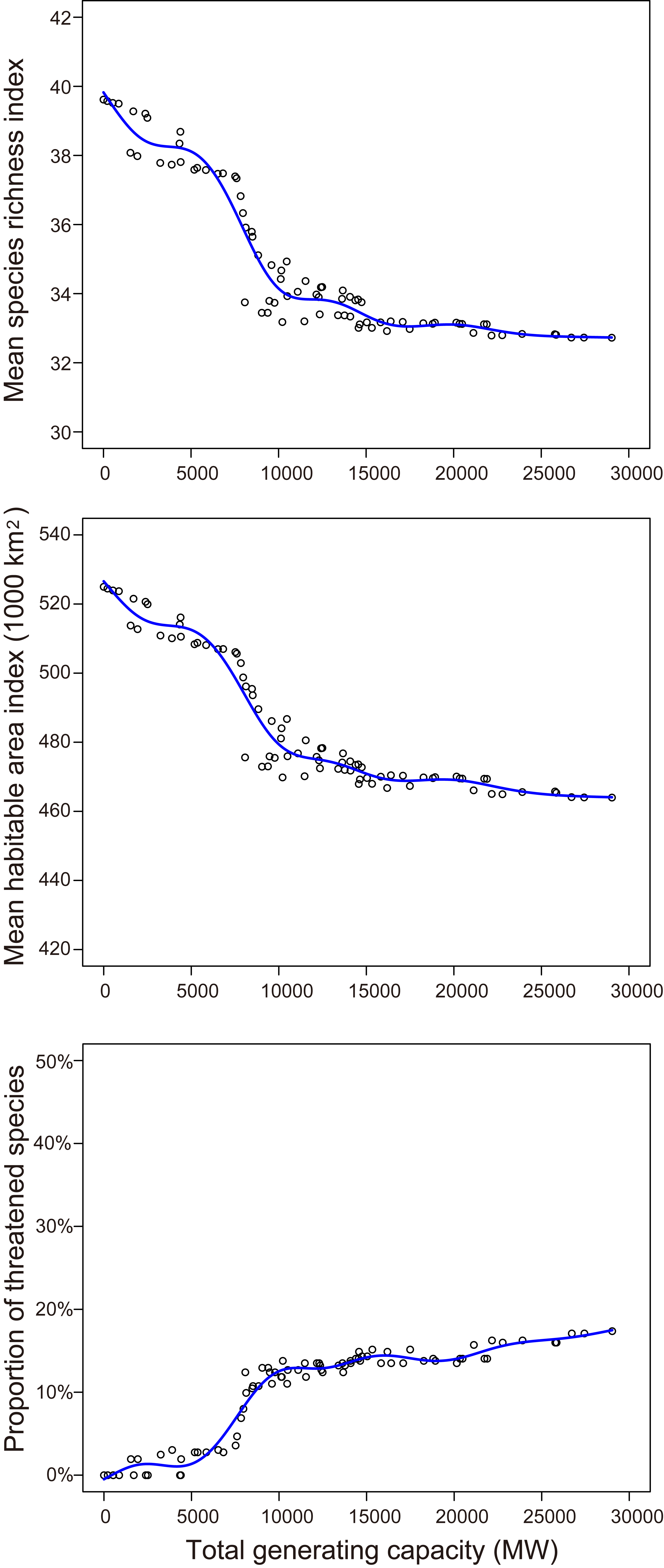

Supplement: S3 Fig — The blue line indicates the spline curve. (TIF) [file pone.0160151.s003.tif]

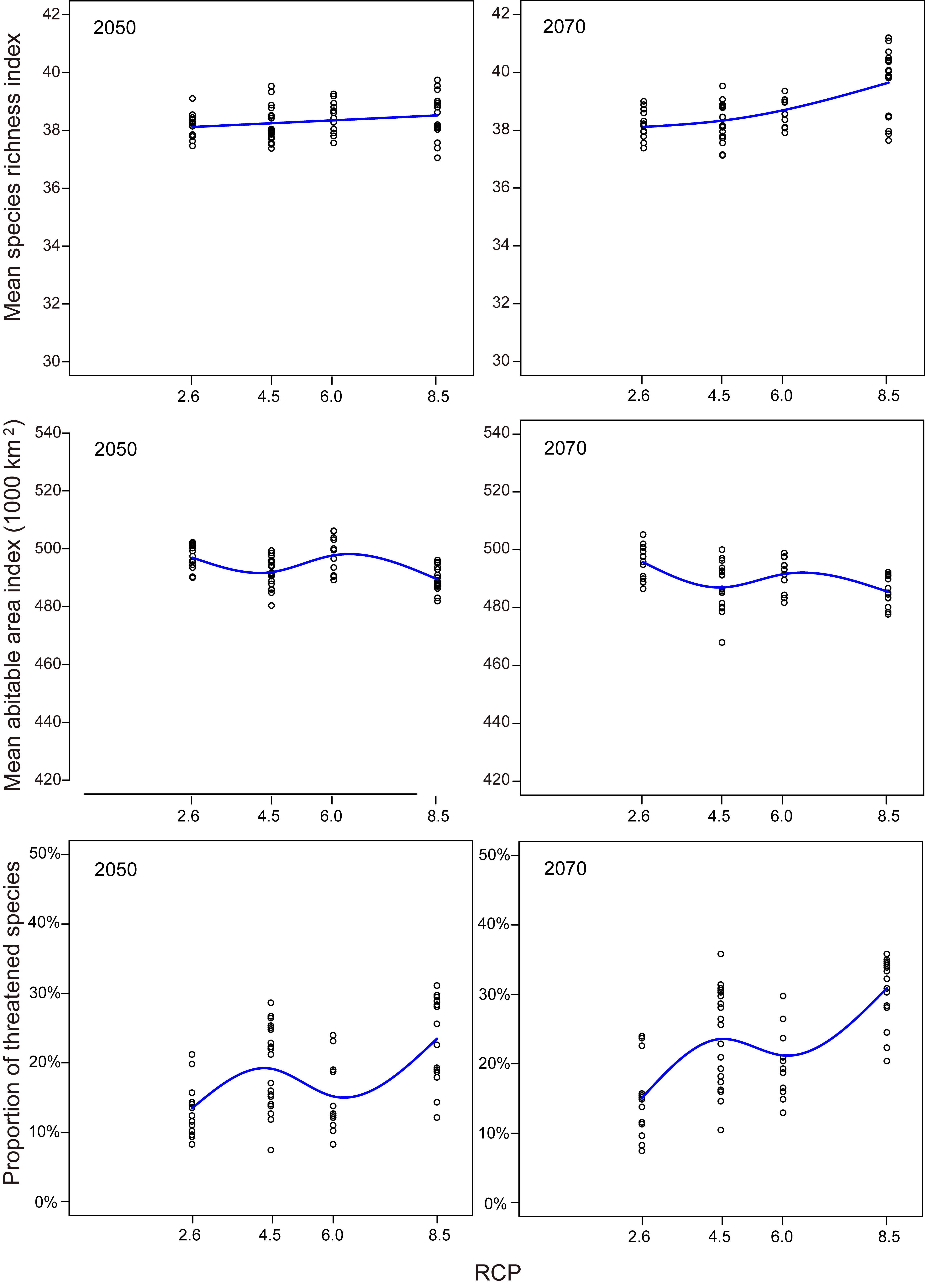

Supplement: S4 Fig — The blue line indicates the spline curve. (TIF) [file pone.0160151.s004.tif]

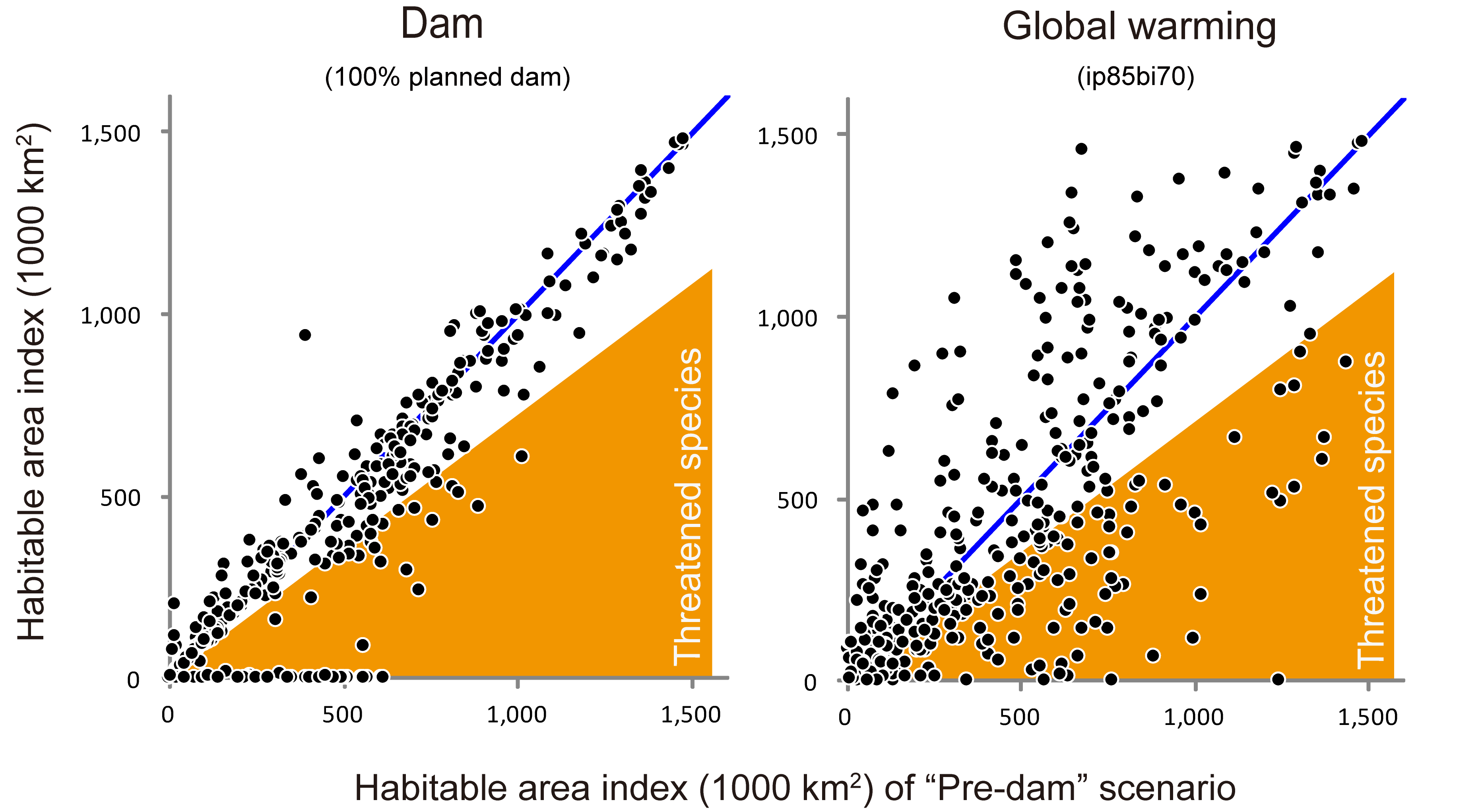

Supplement: S5 Fig — Most fish species showed a contraction in range extent under the planned-dam scenarios (assuming 100% of planned dams were built), while global-warming scenario ip85bi70 had little effect on average habitable area, although the extent of variation was sensitive to species identity. A similar general tendency was apparent in projections from other dam and global-warming scenarios. Solid circles indicate each species and a blue line indicates the threshold of increase/decrease. Species plotted in the orange sector are those that can be considered as threatened. Scenario names in parentheses correspond to those in S2 Table. (TIF) [file pone.0160151.s005.tif]
