# Supplementary material for: Impacts of Dams and Global Warming on Fish Biodiversity in the Indo-Burma Hotspot
Source: PLoS One. 2016 Aug 17;11(8):e0160151. doi: 10.1371/journal.pone.0160151 (PMC4988766; doi:10.1371/journal.pone.0160151)
Supplement: S1 Table — (PDF) [file pone.0160151.s007.pdf]

Species	N. of locations sampled	AUC of Maxent analysis
Acanthocobitis botia	8	0.9746
Acanthopsoides delphax	9	0.8497
Acanthopsoides gracilentus	21	0.9146
Acanthopsoides hapalias	88	0.8563
Acantopsis sp. "Large spot"	61	0.8959
Acantopsis sp. "Small spot"	86	0.8992
Acantopsis sp. "Stripe"	43	0.9011
Acentrogobius viridipunctatus	18	0.9849
Achiroides melanorhijnchus	8	0.981
Akysis ephippifer	5	0.9776
Akysis varius	25	0.947
Ambassis vachellii	31	0.9753
Amblypharyngodon chulabhornae	163	0.7796
Amblyrhynchichthys micracanthus	37	0.9149
Amblyrhynchichthys truncatus	11	0.9953
Anabas testudineus	185	0.755
Annamia normani	10	0.9901
Aplocheilus panchax	71	0.8972
Arius maculatus	11	0.9842
Aulopareia janetae	5	0.9978
Aulopareia unicolor	6	0.9946
Auriglobus nefastus	16	0.9241
Bagarius bagarius	11	0.9766
Bagarius yarrelli	19	0.9472
Bagrichthys majusculus	5	0.8612
Bagrichthys obscurus	11	0.8522
Balitora lancangjiangensis	16	0.9598
Bangana lippa	5	0.9854
Barbichthys laevis	22	0.9273
Barbodes aurotaeniatus	209	0.856
Barbodes rhombeus	51	0.9403
Barbonymus altus	204	0.8502
Barbonymus gonionotus	235	0.8101
Barbonymus schwanefeldii	76	0.8863
Belodontichthys truncatus	16	0.8757
Betta prima	10	0.9691
Betta smaragdina	10	0.9234
Betta splendens	16	0.9381
Boesemania microlepis	25	0.9614
Boleophthalmus boddarti	14	0.9788
Boraras micros	7	0.9841
Boraras urophthalmoides	9	0.9481
Brachirus elongatus	8	0.9806
Brachirus harmandi	65	0.9052
Brachirus siamensis	32	0.9633
Brachydanio albolineata	64	0.9447
Brachydanio rosea	12	0.988
Brachygobius aggregatus	16	0.9355
Brachygobius mekongensis	12	0.9572
Brachygobius sabanus	27	0.9746
Butis butis	23	0.9724
Butis humeralis	9	0.9832
Butis koilomatodon	12	0.9843
Caragobius urolepis	5	0.989
Cephalocassis borneensis	6	0.9409
Channa gachua	242	0.8389
Channa lucius	64	0.8534
Channa micropeltes	7	0.879
Channa striata	277	0.7042
Chaudhuria caudata	25	0.8315
Chitala ornata	10	0.9218
Cirrhinus jullieni	16	0.934
Cirrhinus microlepis	8	0.9767
Cirrhinus prosemion	9	0.8378
Clarias batrachus	38	0.8979
Clarias macrocephalus	10	0.7981
Clarias sp. 1	30	0.926
Clupeichthys aesarnensis	198	0.8198
Clupeichthys goniognathus	77	0.9321
Clupeichthys sp. 1	12	0.9378
Clupeoides borneensis	78	0.8858
Clupisoma sinense	6	0.8464
Coilia lindmani	14	0.954
Corica laciniata	17	0.9543
Cosmochilus harmandi	25	0.9394
Crossocheilus atrilimes	38	0.9024
Crossocheilus oblongus	12	0.8684
Crossocheilus reticulatus	76	0.8623
Crossocheilus sp. 1	5	0.9932
Cyclocheilichthys apogon	168	0.8324
Cyclocheilichthys armatus	62	0.8941
Cyclocheilichthys heteronema	8	0.9635
Cyclocheilichthys lagleri	13	0.9334

Cyclocheilichthys repasson	122	0.8615
Cyclocheilos enoplos	82	0.8622
Cynoglossus feldmanni	12	0.9693
Cynoglossus lingua	9	0.9899
Cynoglossus microlepis	21	0.9722
Cynoglossus puncticeps	18	0.9814
Datnioides polota	5	0.9872
Datnioides undecimradiatus	17	0.9771
Dendrophysa russellii	8	0.9892
Dermogenys siamensis	257	0.7885
Devario aequipinnatus	38	0.9733
Devario apopyris	6	0.9949
Devario laoensis	21	0.9746
Discherodontus ashmeadi	9	0.8733
Doryichthys boaja	105	0.9044
Doryichthys contiguus	16	0.8885
Doryichthys deokhatoides	30	0.9386
Drombus globiceps	11	0.9845
Eleotris melanosoma	27	0.9727
Eleutheronema tetradactylum	16	0.9852
Epalzeorhynchos frenatum	32	0.888
Eugnathogobius kabilia	15	0.94
Eugnathogobius siamensis	41	0.9292
Eugnathogobius variegatus	7	0.9889
Exostoma berdmorei	30	0.9842
Garra cambodgiensis	133	0.9273
Garra fasciacauda	6	0.9401
Garra sp. 1	6	0.8926
Gerres limbatus	19	0.9799
Glossogobius aureus	23	0.9543
Glossogobius giuris	17	0.9807
Glossogobius sparsipapillus	22	0.9827
Glyptothorax buchanani	32	0.9821
Glyptothorax fuscus	8	0.9526
Glyptothorax lampris	24	0.9446
Glyptothorax laosensis	19	0.972
Glyptothorax macromaculatus	10	0.9793
Glyptothorax trilineatus	50	0.9679
Gobiidae indet	6	0.9748
Gobiopsis macrostomus	7	0.9903
Gobiopterus chuno	167	0.8215
Gymnostomus caudimaculatus	22	0.951
Gymnostomus lineatus	13	0.9469
Gymnostomus lobatus	76	0.8912
Gymnostomus ornatipinnis	30	0.9191
Gymnostomus siamensis	224	0.8249
Gyrinocheilus aymonieri	17	0.9552
Gyrinocheilus pennocki	13	0.9468
Hampala dispar	163	0.8486
Hampala macrolepidota	115	0.8469
Helicophagus leptorhynchus	24	0.961
Hemibagrus filamentus	38	0.8767
Hemibagrus nemurus	49	0.8696
Hemibagrus spilopterus	43	0.9428
Hemibagrus wyckioides	11	0.8531
Hemiculterella macrolepis	35	0.9842
Hemigobius hoevenii	10	0.9675
Hippichthys heptagonus	6	0.9812
Homaloptera confuzona	9	0.9681
Homalopteroides smithi	53	0.9053
Homalopteroides tweediei	23	0.8997
Hyporhamphus limbatus	7	0.9666
Hypsibarbus lagleri	7	0.8691
Hypsibarbus malcolmi	57	0.9423
Hypsibarbus vernayi	49	0.9478
Hypsibarbus wetmorei	20	0.9338
Indostomus spinosus	6	0.9775
Johnius borneensis	7	0.9913
Johnius trachycephalus	10	0.9883
Kryptopterus geminus	36	0.9166
Labeo chrysophekadion	49	0.9134
Labeo pierrei	8	0.957
Labiobarbus leptocheilus	115	0.8302
Labiobarbus siamensis	107	0.9138
Laides longibarbis	35	0.8994
Lepidocephalichthys berdmorei	19	0.9561
Lepidocephalichthys furcatus	6	0.9308
Lepidocephalichthys hasselti	112	0.8317
Lepidocephalichthys kranos	16	0.9241
Lepidocephalichthys zeppelini	21	0.9279
Liza macrolepis	11	0.9939
Liza subviridis	35	0.9772
Lobocheilos rhabdoura	41	0.9149
Luciosoma bleekeri	33	0.9317
Lutjanus russellii	6	0.9938
Lycothrissa crocodilus	7	0.9813

Macrochirichthys macrochirus	6	0.8393
Macrognathus semiocellatus	77	0.8481
Macrognathus siamensis	88	0.8743
Macrognathus sp. 1	6	0.9903
Mastacembelus armatus	78	0.9039
Mastacembelus favus	124	0.8379
Mastacembelus tinwini	14	0.9816
Mekongina erythrospila	5	0.9612
Micronema cheveyi	27	0.9316
Monopterus javanensis	98	0.7741
Moolgarda perusii	5	0.9895
Mystacoleucus atridorsalis	64	0.9237
Mystacoleucus chilopterus	6	0.9557
Mystacoleucus ectypus	28	0.9261
Mystacoleucus greenwayi	29	0.9682
Mystacoleucus lepturus	54	0.9736
Mystacoleucus obtusirostris	221	0.7976
Mystacoleucus sp. 1	15	0.9658
Mystus albolineatus	40	0.9312
Mystus atrifasciatus	40	0.9249
Mystus bocourti	9	0.9524
Mystus gulio	24	0.9785
Mystus multiradiatus	8	0.8969
Mystus mysticetus	111	0.8867
Mystus singaringan	17	0.9452
Nandus oxyrhynchus	42	0.9184
Nemacheilus longistriatus	13	0.9778
Nemacheilus pallidus	52	0.8935
Nemacheilus platiceps	31	0.9431
Neosalanx jordani	9	0.9895
Neostethus lankesteri	12	0.988
Notopterus notopterus	156	0.7752
Nuchequula gerreoides	17	0.9819
Oligolepis acutipennis	19	0.9773
Ompok siluroides	74	0.8269
Onychostoma fusiforme	9	0.9934
Onychostoma gerlachi	33	0.9804
Opsarius koratensis	117	0.8609
Opsarius pulchellus	165	0.9246
Oreichthys parvus	6	0.994
Oreoglanis siamensis	39	0.982
Oreoglanis suraswadii	7	0.9929
Oryzias haugiangensis	43	0.9724
Oryzias mekongensis	17	0.9363
Oryzias minutillus	105	0.8474
Oryzias songkhramensis	9	0.992
Osphronemus goramy	15	0.9425
Osteochilus lini	83	0.8755
Osteochilus melanopleura	27	0.8874
Osteochilus microcephalus	66	0.8522
Osteochilus schlegelii	6	0.9846
Osteochilus vittatus	200	0.7794
Osteogeneiosus militaris	9	0.9902
Oxyeleotris marmorata	133	0.7687
Oxyeleotris urophthalmus	16	0.9838
Pangasianodon hypophthalmus	19	0.871
Pangasius bocourti	10	0.8953
Pangasius conchophilus	17	0.955
Pangasius elongatus	8	0.9651
Pangasius larnaudii	9	0.831
Pangasius macronema	75	0.8634
Pangio anguillaris	39	0.8667
Pangio oblonga	13	0.9054
Pao abei	13	0.8729
Pao cambodgiensis	43	0.9145
Pao cochinchinensis	71	0.8846
Pao suvattii	25	0.9391
Pao turgidus	36	0.9602
Papuligobius ocellatus	70	0.9362
Parachela maculicauda	39	0.8436
Parachela oxygastroides	59	0.9188
Parachela siamensis	115	0.8721
Parachela sp. 1	74	0.8627
Parachela williaminae	7	0.9666
Paralaubuca barroni	39	0.923
Paralaubuca riveroi	11	0.9607
Paralaubuca typus	125	0.8682
Parambassis apogonoides	37	0.9277
Parambassis wolffii	47	0.915
Parapocryptes serperaster	13	0.9604
Parasikukia maculata	11	0.954
Periophthalmodon septemradiatus	21	0.982
Periophthalmus chrysospilos	11	0.9857
Periophthalmus gracilis	17	0.9877
Pethia stoliczkana	113	0.944
Phalacronotus apogon	39	0.8832

Phalacronotus bleekeri	7	0.9462
Phenacostethus smithi	53	0.8907
Physoschistura pseudobrunneana	8	0.9821
Physoschistura sp. 1	31	0.9854
Platycephalus indicus	6	0.9904
Plotosus canius	18	0.9747
Polynemus aquilonaris	10	0.9778
Polynemus melanochir	21	0.9834
Poropuntius bantamensis	75	0.9554
Poropuntius carinatus	8	0.9945
Poropuntius laoensis	51	0.9791
Poropuntius normani	82	0.9389
Pristolepis fasciata	261	0.755
Probarbus jullieni	22	0.9682
Pseudapocryptes elongatus	5	0.9818
Pseudobagarius filifer	9	0.9848
Pseudobagarius inermis	6	0.9677
Pseudogobius avicennia	15	0.9787
Pseudogobius javanicus	41	0.9516
Pseudohomaloptera leonardi	47	0.9039
Pseudolais pleurotaenia	20	0.9168
Pseudomystus siamensis	37	0.8722
Puntigrus partipentazona	71	0.846
Puntioplites falcifer	46	0.9134
Puntioplites proctozysron	135	0.8663
Puntius brevis	207	0.7527
Puntius masyai	27	0.9182
Raiamas guttatus	78	0.9121
Rasbora aurotaenia	167	0.8797
Rasbora borapetensis	489	0.7404
Rasbora daniconius	54	0.9414
Rasbora dusonensis	155	0.844
Rasbora paviana	190	0.8731
Rasbora rubrodorsalis	145	0.8765
Rasbora septentrionalis	16	0.9739
Rasbora tornieri	8	0.9371
Rasbora trilineata	97	0.9083
Rasbosoma spilocerca	56	0.8889
Redigobius chrysosoma	16	0.9646
Redigobius nanus	5	0.9897
Rhinogobius mekongianus	74	0.9647
Scaphiodonichthys acanthopterus	39	0.9706
Scaphiodonichthys burmanicus	55	0.9772
Scaphognathops bandanensis	28	0.9689
Scaphognathops stejnegeri	13	0.9522
Scatophagus argus	14	0.9735
Schistura breviceps	51	0.9731
Schistura bucculenta	11	0.9315
Schistura caudofurca	28	0.9892
Schistura clatrata	5	0.9976
Schistura desmotes	5	0.99
Schistura geisleri	5	0.9893
Schistura kengtungensis	28	0.9829
Schistura kongphengi	7	0.9956
Schistura maejotigrina	21	0.9863
Schistura magnifluvis	12	0.9856
Schistura melarancia	13	0.9866
Schistura menanensis	7	0.9918
Schistura namboensis	18	0.9893
Schistura obeini	9	0.978
Schistura poculi	51	0.9703
Schistura porthos	17	0.9905
Schistura pridii	15	0.9888
Schistura sp. 1	15	0.9378
Schistura sp. 2	5	0.9668
Schistura spilota	34	0.9823
Schistura waltoni	30	0.9749
Sectoria atriceps	9	0.9959
Setipinna melanochir	7	0.982
Siganus fuscescens	7	0.9911
Siganus javus	10	0.9857
Sikukia gudgeri	69	0.9485
Sikukia stejnegeri	15	0.9311
Sillago sihama	17	0.9802
Stenogobius mekongensis	18	0.9828
Stigmatogobius minima	10	0.9857
Stigmatogobius pleurostigma	25	0.9788
Stolephorus dubiosus	9	0.9855
Sundasalanx mekongensis	68	0.8657
Syncrossus beauforti	10	0.9613
Syncrossus helodes	57	0.8835
Systomus rubripinnis	151	0.8201
Tenualosa thibaudeaui	18	0.9654
Terapon jarbua	9	0.9733
Thryssocypris tonlesapensis	6	0.9685
Thynnichthys thynnoides	37	0.8795

Tonlesapia tsukawakii	8	0.983
Tor tambra	11	0.9609
Tor tambroides	5	0.9618
Toxotes chatareus	41	0.8724
Trichopodus microlepis	112	0.8962
Trichopodus pectoralis	39	0.8786
Trichopodus trichopterus	386	0.7146
Trichopsis pumila	204	0.8308
Trichopsis schalleri	185	0.8835
Trichopsis vittata	476	0.7215
Trypauchen vagina	8	0.9943
Tuberoschistura cambodgiensis	9	0.9622
Vanmanenia serrilineata	12	0.9769
Wallago attu	6	0.8633
Xenentodon cancila	379	0.7114
Yasuhikotakia eos	16	0.9175
Yasuhikotakia lecontei	45	0.9264
Yasuhikotakia modesta	51	0.9006
Yasuhikotakia morleti	30	0.9165
Zenarchopterus clarus	29	0.9509
Zenarchopterus ectuntio	11	0.9771
Zenarchopterus sp. 1	11	0.9742
