# Supplementary material for: Impacts of Dams and Global Warming on Fish Biodiversity in the Indo-Burma Hotspot
Source: PLoS One. 2016 Aug 17;11(8):e0160151. doi: 10.1371/journal.pone.0160151 (PMC4988766; doi:10.1371/journal.pone.0160151)
Supplement: S2 Table — (PDF) [file pone.0160151.s008.pdf]

Scenario ID	Scenario name	Dam			Global warming		
		Dam construction/removal	Total generating capacity (MW)	*N.* of dams in Indo–Burma region	Global climate model	RCPs	Year
0	Current	Current	7602	596	Current	Current	Current
1	Dam: 5% planned dam	Each planned dam constructed by 5% probability	8075	602	Current	Current	Current
2	Dam: 10% planned dam	Each planned dam constructed by 10% probability	7831	600	Current	Current	Current
3	Dam: 15% planned dam	Each planned dam constructed by 15% probability	10500	614	Current	Current	Current
4	Dam: 20% planned dam	Each planned dam constructed by 20% probability	10220	621	Current	Current	Current
5	Dam: 25% planned dam	Each planned dam constructed by 25% probability	11476	622	Current	Current	Current
6	Dam: 30% planned dam	Each planned dam constructed by 30% probability	14631	631	Current	Current	Current
7	Dam: 35% planned dam	Each planned dam constructed by 35% probability	15327	643	Current	Current	Current
8	Dam: 40% planned dam	Each planned dam constructed by 40% probability	16190	639	Current	Current	Current
9	Dam: 45% planned dam	Each planned dam constructed by 45% probability	15041	640	Current	Current	Current
10	Dam: 50% planned dam	Each planned dam constructed by 50% probability	14568	658	Current	Current	Current
11	Dam: 55% planned dam	Each planned dam constructed by 55% probability	25775	665	Current	Current	Current
12	Dam: 60% planned dam	Each planned dam constructed by 60% probability	22626	670	Current	Current	Current
13	Dam: 65% planned dam	Each planned dam constructed by 65% probability	23905	671	Current	Current	Current
14	Dam: 70% planned dam	Each planned dam constructed by 70% probability	17491	675	Current	Current	Current
15	Dam: 75% planned dam	Each planned dam constructed by 75% probability	22772	688	Current	Current	Current
16	Dam: 80% planned dam	Each planned dam constructed by 80% probability	27348	696	Current	Current	Current
17	Dam: 85% planned dam	Each planned dam constructed by 85% probability	22162	694	Current	Current	Current
18	Dam: 90% planned dam	Each planned dam constructed by 90% probability	27436	706	Current	Current	Current
19	Dam: 95% planned dam	Each planned dam constructed by 95% probability	26716	712	Current	Current	Current
20	Dam: 100% planned dam	Each planned dam constructed by 100% probability	29027	717	Current	Current	Current
21	Dam: 5% mainstream dam	Each planned lower Mekong mainstream dam constructed by 5% probability	9472	597	Current	Current	Current
22	Dam: 10% mainstream dam	Each planned lower Mekong mainstream dam constructed by 10% probability	9041	598	Current	Current	Current
23	Dam: 15% mainstream dam	Each planned lower Mekong mainstream dam constructed by 15% probability	8832	597	Current	Current	Current
24	Dam: 20% mainstream dam	Each planned lower Mekong mainstream dam constructed by 20% probability	9377	599	Current	Current	Current
25	Dam: 25% mainstream dam	Each planned lower Mekong mainstream dam constructed by 25% probability	9769	598	Current	Current	Current
26	Dam: 30% mainstream dam	Each planned lower Mekong mainstream dam constructed by 30% probability	16401	601	Current	Current	Current
27	Dam: 35% mainstream dam	Each planned lower Mekong mainstream dam constructed by 35% probability	12349	600	Current	Current	Current
28	Dam: 40% mainstream dam	Each planned lower Mekong mainstream dam constructed by 40% probability	13391	599	Current	Current	Current
29	Dam: 45% mainstream dam	Each planned lower Mekong mainstream dam constructed by 45% probability	13763	603	Current	Current	Current
30	Dam: 50% mainstream dam	Each planned lower Mekong mainstream dam constructed by 50% probability	18265	605	Current	Current	Current
31	Dam: 55% mainstream dam	Each planned lower Mekong mainstream dam constructed by 55% probability	17092	602	Current	Current	Current
32	Dam: 60% mainstream dam	Each planned lower Mekong mainstream dam constructed by 60% probability	14098	602	Current	Current	Current
33	Dam: 65% mainstream dam	Each planned lower Mekong mainstream dam constructed by 65% probability	15828	603	Current	Current	Current
34	Dam: 70% mainstream dam	Each planned lower Mekong mainstream dam constructed by 70% probability	18800	606	Current	Current	Current
35	Dam: 75% mainstream dam	Each planned lower Mekong mainstream dam constructed by 75% probability	20154	606	Current	Current	Current
36	Dam: 80% mainstream dam	Each planned lower Mekong mainstream dam constructed by 80% probability	20333	607	Current	Current	Current
37	Dam: 85% mainstream dam	Each planned lower Mekong mainstream dam constructed by 85% probability	18923	606	Current	Current	Current
38	Dam: 90% mainstream dam	Each planned lower Mekong mainstream dam constructed by 90% probability	20480	608	Current	Current	Current
39	Dam: 95% mainstream dam	Each planned lower Mekong mainstream dam constructed by 95% probability	21735	608	Current	Current	Current
40	Dam: 100% mainstream dam	Each planned lower Mekong mainstream dam constructed by 100% probability	21890	609	Current	Current	Current
41	Dam: 5% tributary dam	Each planned non–Mekong mainstream dam constructed by 5% probability	7966	601	Current	Current	Current
42	Dam: 10% tributary dam	Each planned non–Mekong mainstream dam constructed by 10% probability	8121	611	Current	Current	Current
43	Dam: 15% tributary dam	Each planned non–Mekong mainstream dam constructed by 15% probability	8509	613	Current	Current	Current
44	Dam: 20% tributary dam	Each planned non–Mekong mainstream dam constructed by 20% probability	8473	616	Current	Current	Current
45	Dam: 25% tributary dam	Each planned non–Mekong mainstream dam constructed by 25% probability	9595	629	Current	Current	Current
46	Dam: 30% tributary dam	Each planned non–Mekong mainstream dam constructed by 30% probability	10157	633	Current	Current	Current
47	Dam: 35% tributary dam	Each planned non–Mekong mainstream dam constructed by 35% probability	10125	635	Current	Current	Current
48	Dam: 40% tributary dam	Each planned non–Mekong mainstream dam constructed by 40% probability	10460	647	Current	Current	Current
49	Dam: 45% tributary dam	Each planned non–Mekong mainstream dam constructed by 45% probability	11091	644	Current	Current	Current
50	Dam: 50% tributary dam	Each planned non–Mekong mainstream dam constructed by 50% probability	11543	653	Current	Current	Current
51	Dam: 55% tributary dam	Each planned non–Mekong mainstream dam constructed by 55% probability	12296	657	Current	Current	Current
52	Dam: 60% tributary dam	Each planned non–Mekong mainstream dam constructed by 60% probability	12493	666	Current	Current	Current
53	Dam: 65% tributary dam	Each planned non–Mekong mainstream dam constructed by 65% probability	12162	667	Current	Current	Current
54	Dam: 70% tributary dam	Each planned non–Mekong mainstream dam constructed by 70% probability	12417	674	Current	Current	Current
55	Dam: 75% tributary dam	Each planned non–Mekong mainstream dam constructed by 75% probability	13666	674	Current	Current	Current
56	Dam: 80% tributary dam	Each planned non–Mekong mainstream dam constructed by 80% probability	13622	679	Current	Current	Current
57	Dam: 85% tributary dam	Each planned non–Mekong mainstream dam constructed by 85% probability	14076	687	Current	Current	Current
58	Dam: 90% tributary dam	Each planned non–Mekong mainstream dam constructed by 90% probability	14384	691	Current	Current	Current
59	Dam: 95% tributary dam	Each planned non–Mekong mainstream dam constructed by 95% probability	14540	700	Current	Current	Current
60	Dam: 100% tributary dam	Each planned non–Mekong mainstream dam constructed by 100% probability	14738	704	Current	Current	Current
61	Dam: 5% dam removal	Each existing dam removed by 5% probability	7522	584	Current	Current	Current
62	Dam: 10% dam removal	Each existing dam removed by 10% probability	6825	525	Current	Current	Current
63	Dam: 15% dam removal	Each existing dam removed by 15% probability	6526	494	Current	Current	Current
64	Dam: 20% dam removal	Each existing dam removed by 20% probability	5189	468	Current	Current	Current
65	Dam: 25% dam removal	Each existing dam removed by 25% probability	5852	460	Current	Current	Current
66	Dam: 30% dam removal	Each existing dam removed by 30% probability	5354	403	Current	Current	Current
67	Dam: 35% dam removal	Each existing dam removed by 35% probability	4389	383	Current	Current	Current
68	Dam: 40% dam removal	Each existing dam removed by 40% probability	3899	362	Current	Current	Current
69	Dam: 45% dam removal	Each existing dam removed by 45% probability	4408	336	Current	Current	Current
70	Dam: 50% dam removal	Each existing dam removed by 50% probability	3241	287	Current	Current	Current
71	Dam: 55% dam removal	Each existing dam removed by 55% probability	4344	371	Current	Current	Current
72	Dam: 60% dam removal	Each existing dam removed by 60% probability	2508	240	Current	Current	Current
73	Dam: 65% dam removal	Each existing dam removed by 65% probability	2390	230	Current	Current	Current
74	Dam: 70% dam removal	Each existing dam removed by 70% probability	1933	158	Current	Current	Current
75	Dam: 75% dam removal	Each existing dam removed by 75% probability	1700	142	Current	Current	Current
76	Dam: 80% dam removal	Each existing dam removed by 80% probability	1540	108	Current	Current	Current
77	Dam: 85% dam removal	Each existing dam removed by 85% probability	871	91	Current	Current	Current
78	Dam: 90% dam removal	Each existing dam removed by 90% probability	527	48	Current	Current	Current
79	Dam: 95% dam removal	Each existing dam removed by 95% probability	212	24	Current	Current	Current
80	Dam: 100% dam removal (Pre–dam scenario)	Each existing dam removed by 100% probability	0	0	Current	Current	Current
81	Global warming: ac45bi50	Current	7602	596	ACCESS1–0	4.5	2050
82	Global warming: ac45bi70	Current	7602	596	ACCESS1–0	4.5	2070
83	Global warming: ac85bi50	Current	7602	596	ACCESS1–0	8.5	2050
84	Global warming: ac85bi70	Current	7602	596	ACCESS1–0	8.5	2070
85	Global warming: bc26bi50	Current	7602	596	BCC–CSM1–1	2.6	2050
86	Global warming: bc26bi70	Current	7602	596	BCC–CSM1–1	2.6	2070
87	Global warming: bc45bi50	Current	7602	596	BCC–CSM1–1	4.5	2050
88	Global warming: bc45bi70	Current	7602	596	BCC–CSM1–1	4.5	2070
89	Global warming: bc60bi50	Current	7602	596	BCC–CSM1–1	6.0	2050
90	Global warming: bc60bi70	Current	7602	596	BCC–CSM1–1	6.0	2070
91	Global warming: bc85bi50	Current	7602	596	BCC–CSM1–1	8.5	2050
92	Global warming: bc85bi70	Current	7602	596	BCC–CSM1–1	8.5	2070
93	Global warming: cc26bi50	Current	7602	596	CCSM4	2.6	2050
94	Global warming: cc26bi70	Current	7602	596	CCSM4	2.6	2070
95	Global warming: cc45bi50	Current	7602	596	CCSM4	4.5	2050
96	Global warming: cc45bi70	Current	7602	596	CCSM4	4.5	2070
97	Global warming: cc60bi50	Current	7602	596	CCSM4	6.0	2050
98	Global warming: cc60bi70	Current	7602	596	CCSM4	6.0	2070
99	Global warming: cc85bi50	Current	7602	596	CCSM4	8.5	2050
100	Global warming: cc85bi70	Current	7602	596	CCSM4	8.5	2070
101	Global warming: ce45bi50	Current	7602	596	CESM1–CAM5–1–FV2	4.5	2050
102	Global warming: ce45bi70	Current	7602	596	CESM1–CAM5–1–FV2	4.5	2070
103	Global warming: cn26bi50	Current	7602	596	CNRM–CM5	2.6	2050
104	Global warming: cn26bi70	Current	7602	596	CNRM–CM5	2.6	2070
105	Global warming: cn45bi50	Current	7602	596	CNRM–CM5	4.5	2050
106	Global warming: cn45bi70	Current	7602	596	CNRM–CM5	4.5	2070
107	Global warming: cn85bi50	Current	7602	596	CNRM–CM5	8.5	2050
108	Global warming: cn85bi70	Current	7602	596	CNRM–CM5	8.5	2070
109	Global warming: gd26bi50	Current	7602	596	GFDL–ESM2G	2.6	2050
110	Global warming: gd26bi70	Current	7602	596	GFDL–ESM2G	2.6	2070
111	Global warming: gd45bi50	Current	7602	596	GFDL–ESM2G	4.5	2050
112	Global warming: gd45bi70	Current	7602	596	GFDL–ESM2G	4.5	2070
113	Global warming: gd60bi50	Current	7602	596	GFDL–ESM2G	6.0	2050
114	Global warming: gd60bi70	Current	7602	596	GFDL–ESM2G	6.0	2070
115	Global warming: gf26bi50	Current	7602	596	GFDL–CM3	2.6	2050
116	Global warming: gf26bi70	Current	7602	596	GFDL–CM3	2.6	2070
117	Global warming: gf45bi50	Current	7602	596	GFDL–CM3	4.5	2050
118	Global warming: gf45bi70	Current	7602	596	GFDL–CM3	4.5	2070
119	Global warming: gf85bi50	Current	7602	596	GFDL–CM3	8.5	2050
120	Global warming: gf85bi70	Current	7602	596	GFDL–CM3	8.5	2070
121	Global warming: gs26bi50	Current	7602	596	GISS–E2–R	2.6	2050
122	Global warming: gs26bi70	Current	7602	596	GISS–E2–R	2.6	2070
123	Global warming: gs45bi50	Current	7602	596	GISS–E2–R	4.5	2050
124	Global warming: gs45bi70	Current	7602	596	GISS–E2–R	4.5	2070
125	Global warming: gs60bi50	Current	7602	596	GISS–E2–R	6.0	2050
126	Global warming: gs60bi70	Current	7602	596	GISS–E2–R	6.0	2070
127	Global warming: gs85bi50	Current	7602	596	GISS–E2–R	8.5	2050
128	Global warming: gs85bi70	Current	7602	596	GISS–E2–R	8.5	2070
129	Global warming: hd26bi50	Current	7602	596	HadGEM2–AO	2.6	2050
130	Global warming: hd26bi70	Current	7602	596	HadGEM2–AO	2.6	2070
131	Global warming: hd45bi50	Current	7602	596	HadGEM2–AO	4.5	2050
132	Global warming: hd45bi70	Current	7602	596	HadGEM2–AO	4.5	2070
133	Global warming: hd60bi50	Current	7602	596	HadGEM2–AO	6.0	2050
134	Global warming: hd60bi70	Current	7602	596	HadGEM2–AO	6.0	2070
135	Global warming: hd85bi50	Current	7602	596	HadGEM2–AO	8.5	2050
136	Global warming: hd85bi70	Current	7602	596	HadGEM2–AO	8.5	2070
137	Global warming: he26bi50	Current	7602	596	HadGEM2–ES	2.6	2050
138	Global warming: he26bi70	Current	7602	596	HadGEM2–ES	2.6	2070
139	Global warming: he45bi50	Current	7602	596	HadGEM2–ES	4.5	2050
140	Global warming: he45bi70	Current	7602	596	HadGEM2–ES	4.5	2070

141	Global warming: he60bi50	Current	7602	596	HadGEM2-ES	6.0	2050
142	Global warming: he60bi70	Current	7602	596	HadGEM2-ES	6.0	2070
143	Global warming: he85bi50	Current	7602	596	HadGEM2-ES	8.5	2050
144	Global warming: he85bi70	Current	7602	596	HadGEM2-ES	8.5	2070
145	Global warming: hg45bi50	Current	7602	596	HadGEM2-CC	4.5	2050
146	Global warming: hg45bi70	Current	7602	596	HadGEM2-CC	4.5	2070
147	Global warming: hg85bi50	Current	7602	596	HadGEM2-CC	8.5	2050
148	Global warming: hg85bi70	Current	7602	596	HadGEM2-CC	8.5	2070
149	Global warming: in45bi50	Current	7602	596	INMCM4	4.5	2050
150	Global warming: in45bi70	Current	7602	596	INMCM4	4.5	2070
151	Global warming: in85bi50	Current	7602	596	INMCM4	8.5	2050
152	Global warming: in85bi70	Current	7602	596	INMCM4	8.5	2070
153	Global warming: ip26bi50	Current	7602	596	IPSL-CM5A-LR	2.6	2050
154	Global warming: ip26bi70	Current	7602	596	IPSL-CM5A-LR	2.6	2070
155	Global warming: ip45bi50	Current	7602	596	IPSL-CM5A-LR	4.5	2050
156	Global warming: ip45bi70	Current	7602	596	IPSL-CM5A-LR	4.5	2070
157	Global warming: ip60bi50	Current	7602	596	IPSL-CM5A-LR	6.0	2050
158	Global warming: ip60bi70	Current	7602	596	IPSL-CM5A-LR	6.0	2070
159	Global warming: ip85bi50	Current	7602	596	IPSL-CM5A-LR	8.5	2050
160	Global warming: ip85bi70	Current	7602	596	IPSL-CM5A-LR	8.5	2070
161	Global warming: mc26bi50	Current	7602	596	MIROC5	2.6	2050
162	Global warming: mc26bi70	Current	7602	596	MIROC5	2.6	2070
163	Global warming: mc45bi50	Current	7602	596	MIROC5	4.5	2050
164	Global warming: mc45bi70	Current	7602	596	MIROC5	4.5	2070
165	Global warming: mc60bi50	Current	7602	596	MIROC5	6.0	2050
166	Global warming: mc60bi70	Current	7602	596	MIROC5	6.0	2070
167	Global warming: mc85bi50	Current	7602	596	MIROC5	8.5	2050
168	Global warming: mc85bi70	Current	7602	596	MIROC5	8.5	2070
169	Global warming: mg26bi50	Current	7602	596	MRI-CGCM3	2.6	2050
170	Global warming: mg26bi70	Current	7602	596	MRI-CGCM3	2.6	2070
171	Global warming: mg45bi50	Current	7602	596	MRI-CGCM3	4.5	2050
172	Global warming: mg45bi70	Current	7602	596	MRI-CGCM3	4.5	2070
173	Global warming: mg60bi50	Current	7602	596	MRI-CGCM3	6.0	2050
174	Global warming: mg60bi70	Current	7602	596	MRI-CGCM3	6.0	2070
175	Global warming: mg85bi50	Current	7602	596	MRI-CGCM3	8.5	2050
176	Global warming: mg85bi70	Current	7602	596	MRI-CGCM3	8.5	2070
177	Global warming: mi26bi50	Current	7602	596	MIROC-ESM-CHEM	2.6	2050
178	Global warming: mi26bi70	Current	7602	596	MIROC-ESM-CHEM	2.6	2070
179	Global warming: mi45bi50	Current	7602	596	MIROC-ESM-CHEM	4.5	2050
180	Global warming: mi45bi70	Current	7602	596	MIROC-ESM-CHEM	4.5	2070
181	Global warming: mi60bi50	Current	7602	596	MIROC-ESM-CHEM	6.0	2050
182	Global warming: mi60bi70	Current	7602	596	MIROC-ESM-CHEM	6.0	2070
183	Global warming: mi85bi50	Current	7602	596	MIROC-ESM-CHEM	8.5	2050
184	Global warming: mi85bi70	Current	7602	596	MIROC-ESM-CHEM	8.5	2070
185	Global warming: mp26bi50	Current	7602	596	MPI-ESM-LR	2.6	2050
186	Global warming: mp26bi70	Current	7602	596	MPI-ESM-LR	2.6	2070
187	Global warming: mp45bi50	Current	7602	596	MPI-ESM-LR	4.5	2050
188	Global warming: mp45bi70	Current	7602	596	MPI-ESM-LR	4.5	2070
189	Global warming: mp85bi50	Current	7602	596	MPI-ESM-LR	8.5	2050
190	Global warming: mp85bi70	Current	7602	596	MPI-ESM-LR	8.5	2070
191	Global warming: mr26bi50	Current	7602	596	MIROC-ESM	2.6	2050
192	Global warming: mr26bi70	Current	7602	596	MIROC-ESM	2.6	2070
193	Global warming: mr45bi50	Current	7602	596	MIROC-ESM	4.5	2050
194	Global warming: mr45bi70	Current	7602	596	MIROC-ESM	4.5	2070
195	Global warming: mr60bi50	Current	7602	596	MIROC-ESM	6.0	2050
196	Global warming: mr60bi70	Current	7602	596	MIROC-ESM	6.0	2070
197	Global warming: mr85bi50	Current	7602	596	MIROC-ESM	8.5	2050
198	Global warming: mr85bi70	Current	7602	596	MIROC-ESM	8.5	2070
199	Global warming: no26bi50	Current	7602	596	NorESM1-M	2.6	2050
200	Global warming: no26bi70	Current	7602	596	NorESM1-M	2.6	2070
201	Global warming: no45bi50	Current	7602	596	NorESM1-M	4.5	2050
202	Global warming: no45bi70	Current	7602	596	NorESM1-M	4.5	2070
203	Global warming: no60bi50	Current	7602	596	NorESM1-M	6.0	2050
204	Global warming: no60bi70	Current	7602	596	NorESM1-M	6.0	2070
205	Global warming: no85bi50	Current	7602	596	NorESM1-M	8.5	2050
206	Global warming: no85bi70	Current	7602	596	NorESM1-M	8.5	2070
207	Additive: Current dam + ce45bi50	Current	7602	596	CESM1-CAM5-1-FV2	4.5	2050
208	Additive: 5% planned dam + gd26bi70	Each planned dam constructed by 5% probability	8075	602	GFDL-ESM2G	2.6	2070
209	Additive: 10% planned dam + cc45bi70	Each planned dam constructed by 10% probability	7831	600	CCSM4	4.5	2070
210	Additive: 15% planned dam + mc60bi50	Each planned dam constructed by 15% probability	10500	614	MIROC5	6.0	2050
211	Additive: 20% planned dam + bc45bi70	Each planned dam constructed by 20% probability	10220	621	BCC-CSM1-1	4.5	2070
212	Additive: 25% planned dam + gs45bi50	Each planned dam constructed by 25% probability	11476	622	GISS-E2-R	4.5	2050
213	Additive: 30% planned dam + mr26bi70	Each planned dam constructed by 30% probability	14631	631	MIROC-ESM	2.6	2070
214	Additive: 35% planned dam + gf85bi70	Each planned dam constructed by 35% probability	15327	643	GFDL-CM3	8.5	2070
215	Additive: 40% planned dam + gf45bi50	Each planned dam constructed by 40% probability	16190	639	GFDL-CM3	4.5	2050
216	Additive: 45% planned dam + hg45bi70	Each planned dam constructed by 45% probability	15041	640	HadGEM2-CC	4.5	2070
217	Additive: 50% planned dam + in85bi50	Each planned dam constructed by 50% probability	14568	658	INMCM4	8.5	2050
218	Additive: 55% planned dam + hg85bi70	Each planned dam constructed by 55% probability	25775	665	HadGEM2-CC	8.5	2070
219	Additive: 60% planned dam + he45bi70	Each planned dam constructed by 60% probability	22626	670	HadGEM2-ES	4.5	2070
220	Additive: 65% planned dam + hd26bi50	Each planned dam constructed by 65% probability	23905	671	HadGEM2-AO	2.6	2050
221	Additive: 70% planned dam + cc60bi70	Each planned dam constructed by 70% probability	17491	675	CCSM4	6.0	2070
222	Additive: 75% planned dam + mc26bi50	Each planned dam constructed by 75% probability	22772	688	MIROC5	2.6	2050
223	Additive: 80% planned dam + he85bi70	Each planned dam constructed by 80% probability	27348	696	HadGEM2-ES	8.5	2070
224	Additive: 85% planned dam + cn26bi50	Each planned dam constructed by 85% probability	22162	694	CNRM-CM5	2.6	2050
225	Additive: 90% planned dam + ce45bi70	Each planned dam constructed by 90% probability	27436	706	CESM1-CAM5-1-FV2	4.5	2070
226	Additive: 95% planned dam + he60bi50	Each planned dam constructed by 95% probability	26716	712	HadGEM2-ES	6.0	2050
227	Additive: 100% planned dam + ce45bi50	Each planned dam constructed by 100% probability	29027	717	CESM1-CAM5-1-FV2	4.5	2050
228	Additive: 5% mainstream dam + mi85bi50	Each planned lower Mekong mainstream dam constructed by 5% probability	9472	597	MIROC-ESM-CHEM	8.5	2050
229	Additive: 10% mainstream dam + mg85bi50	Each planned lower Mekong mainstream dam constructed by 10% probability	9041	598	MRI-CGCM3	8.5	2050
230	Additive: 15% mainstream dam + no60bi70	Each planned lower Mekong mainstream dam constructed by 15% probability	8832	597	NorESM1-M	6.0	2070
231	Additive: 20% mainstream dam + gf45bi70	Each planned lower Mekong mainstream dam constructed by 20% probability	9377	599	GFDL-CM3	4.5	2070
232	Additive: 25% mainstream dam + he26bi70	Each planned lower Mekong mainstream dam constructed by 25% probability	9769	598	HadGEM2-ES	2.6	2070
233	Additive: 30% mainstream dam + mi60bi70	Each planned lower Mekong mainstream dam constructed by 30% probability	16401	601	MIROC-ESM-CHEM	6.0	2070
234	Additive: 35% mainstream dam + no26bi70	Each planned lower Mekong mainstream dam constructed by 35% probability	12349	600	NorESM1-M	2.6	2070
235	Additive: 40% mainstream dam + he45bi50	Each planned lower Mekong mainstream dam constructed by 40% probability	13391	599	HadGEM2-ES	4.5	2050
236	Additive: 45% mainstream dam + mg85bi70	Each planned lower Mekong mainstream dam constructed by 45% probability	13763	603	MRI-CGCM3	8.5	2070
237	Additive: 50% mainstream dam + gd60bi70	Each planned lower Mekong mainstream dam constructed by 50% probability	18265	605	GFDL-ESM2G	6.0	2070
238	Additive: 55% mainstream dam + cc85bi70	Each planned lower Mekong mainstream dam constructed by 55% probability	17092	602	CCSM4	8.5	2070
239	Additive: 60% mainstream dam + no60bi50	Each planned lower Mekong mainstream dam constructed by 60% probability	14098	602	NorESM1-M	6.0	2050
240	Additive: 65% mainstream dam + hd26bi70	Each planned lower Mekong mainstream dam constructed by 65% probability	15828	603	HadGEM2-AO	2.6	2070
241	Additive: 70% mainstream dam + he60bi70	Each planned lower Mekong mainstream dam constructed by 70% probability	18800	606	HadGEM2-ES	6.0	2070
242	Additive: 75% mainstream dam + mi26bi70	Each planned lower Mekong mainstream dam constructed by 75% probability	20154	606	MIROC-ESM-CHEM	2.6	2070
243	Additive: 80% mainstream dam + mr85bi70	Each planned lower Mekong mainstream dam constructed by 80% probability	20333	607	MIROC-ESM	8.5	2070
244	Additive: 85% mainstream dam + cc45bi50	Each planned lower Mekong mainstream dam constructed by 85% probability	18923	606	CCSM4	4.5	2050
245	Additive: 90% mainstream dam + gs85bi50	Each planned lower Mekong mainstream dam constructed by 90% probability	20480	608	GISS-E2-R	8.5	2050
246	Additive: 95% mainstream dam + cc26bi70	Each planned lower Mekong mainstream dam constructed by 95% probability	21735	608	CCSM4	2.6	2070
247	Additive: 100% mainstream dam + gf85bi50	Each planned lower Mekong mainstream dam constructed by 100% probability	21890	609	GFDL-CM3	8.5	2050
248	Additive: 5% tributary dam + mc85bi70	Each planned non-Mekong mainstream dam constructed by 5% probability	7966	601	MIROC5	8.5	2070
249	Additive: 10% tributary dam + mg45bi50	Each planned non-Mekong mainstream dam constructed by 10% probability	8121	611	MRI-CGCM3	4.5	2050
250	Additive: 15% tributary dam + ip45bi70	Each planned non-Mekong mainstream dam constructed by 15% probability	8509	613	IPSL-CM5A-LR	4.5	2070
251	Additive: 20% tributary dam + mc45bi50	Each planned non-Mekong mainstream dam constructed by 20% probability	8473	616	MIROC5	4.5	2050
252	Additive: 25% tributary dam + no85bi50	Each planned non-Mekong mainstream dam constructed by 25% probability	9595	629	NorESM1-M	8.5	2050
253	Additive: 30% tributary dam + mg26bi50	Each planned non-Mekong mainstream dam constructed by 30% probability	10157	633	MRI-CGCM3	2.6	2050
254	Additive: 35% tributary dam + hd45bi50	Each planned non-Mekong mainstream dam constructed by 35% probability	10125	635	HadGEM2-AO	4.5	2050
255	Additive: 40% tributary dam + in45bi70	Each planned non-Mekong mainstream dam constructed by 40% probability	10460	647	INMCM4	4.5	2070
256	Additive: 45% tributary dam + ac45bi50	Each planned non-Mekong mainstream dam constructed by 45% probability	11091	644	ACCESS1-0	4.5	2050
257	Additive: 50% tributary dam + gd45bi70	Each planned non-Mekong mainstream dam constructed by 50% probability	11543	653	GFDL-ESM2G	4.5	2070
258	Additive: 55% tributary dam + gs26bi50	Each planned non-Mekong mainstream dam constructed by 55% probability	12296	657	GISS-E2-R	2.6	2050
259	Additive: 60% tributary dam + mr26bi50	Each planned non-Mekong mainstream dam constructed by 60% probability	12493	666	MIROC-ESM	2.6	2050
260	Additive: 65% tributary dam + gd45bi50	Each planned non-Mekong mainstream dam constructed by 65% probability	12162	667	GFDL-ESM2G	4.5	2050
261	Additive: 70% tributary dam + mr85bi50	Each planned non-Mekong mainstream dam constructed by 70% probability	12417	674	MIROC-ESM	8.5	2050
262	Additive: 75% tributary dam + bc26bi70	Each planned non-Mekong mainstream dam constructed by 75% probability	13666	674	BCC-CSM1-1	2.6	2070
263	Additive: 80% tributary dam + bc26bi70	Each planned non-Mekong mainstream dam constructed by 80% probability	13622	679	BCC-CSM1-1	2.6	2070
264	Additive: 85% tributary dam + no45bi50	Each planned non-Mekong mainstream dam constructed by 85% probability	14076	687	NorESM1-M	4.5	2050
265	Additive: 90% tributary dam + bc85bi70	Each planned non-Mekong mainstream dam constructed by 90% probability	14384	691	BCC-CSM1-1	8.5	2070
266	Additive: 95% tributary dam + ip26bi50	Each planned non-Mekong mainstream dam constructed by 95% probability	14540	700	IPSL-CM5A-LR	2.6	2050
267	Additive: 100% tributary dam + mp26bi70	Each planned non-Mekong mainstream dam constructed by 100% probability	14738	704	MPI-ESM-LR	2.6	2070
268	Additive: 5% dam removal + hd60bi50	Each existing dam removed by 5% probability	7522	584	HadGEM2-AO	6.0	2050
269	Additive: 10% dam removal + ip85bi70	Each existing dam removed by 10% probability	6825	525	IPSL-CM5A-LR	8.5	2070
270	Additive: 15% dam removal + he85bi50	Each existing dam removed by 15% probability	6526	494	HadGEM2-ES	8.5	2050
271	Additive: 20% dam removal + mg45bi70	Each existing dam removed by 20% probability	5189	468	MRI-CGCM3	4.5	2070
272	Additive: 25% dam removal + ac45bi70	Each existing dam removed by 25% probability	5852	460	ACCESS1-0	4.5	2070
273	Additive: 30% dam removal + mc60bi70	Each existing dam removed by 30% probability	5354	403	MIROC5	6.0	2070
274	Additive: 35% dam removal + gd60bi50	Each existing dam removed by 35% probability	4389	383	GFDL-ESM2G	6.0	2050
275	Additive: 40% dam removal + no85bi70	Each existing dam removed by 40% probability	3899	362	NorESM1-M	8.5	2070
276	Additive: 45% dam removal + ip60bi70	Each existing dam removed by 45% probability	4408	336	IPSL-CM5A-LR	6.0	2070
277	Additive: 50% dam removal + mp85bi70	Each existing dam removed by 50% probability	3241	287	MPI-ESM-LR	8.5	2070
278	Additive: 55% dam removal + mc85bi50	Each existing dam removed by 55% probability	4344	371	MIROC5	8.5	2050
279	Additive: 60% dam removal + mg26bi70	Each existing dam removed by 60% probability	2508	240	MRI-CGCM3	2.6	2070
280	Additive: 65% dam removal + mr60bi70	Each existing dam removed by 65% probability	2390	230	MIROC-ESM	6.0	2070
281	Additive: 70% dam removal + ip26bi70	Each existing dam removed by 70% probability	1933	158	IPSL-CM5A-LR	2.6	2070
282	Additive: 75% dam removal + ip85bi50	Each existing dam removed by 75% probability	1700	142	IPSL-CM5A-LR	8.5	2050
283	Additive: 80% dam removal + mc45bi70	Each existing dam removed by 80% probability	1540	108	MIROC5	4.5	2070
284	Additive: 85% dam removal + hg45bi50	Each existing dam removed by 85% probability	871	91	HadGEM2-CC	4.5	2050
285	Additive: 90% dam removal + mg60bi50	Each existing dam removed by 90% probability	527	48	MRI-CGCM3	6.0	2050

286	Additive: 95% dam removal + mp45bi50	Each existing dam removed by 95% probability	212	24	MPI−ESM−LR	4.5	2050
287	Additive: 100% dam removal + mp26bi50	Each existing dam removed by 100% probability	0	0	MPI−ESM−LR	2.6	2050
288	Synergy: Current dam + mp26bi50 + synergistic effect	Current	7602	596	MPI−ESM−LR	2.6	2050
289	Synergy: 5% planned dam + gs60bi50 + synergistic effect	Each planned dam constructed by 5% probability	8075	602	GISS−E2−R	6.0	2050
290	Synergy: 10% planned dam + ce45bi50 + synergistic effect	Each planned dam constructed by 10% probability	7831	600	CESM1−CAM5−1−FV2	4.5	2050
291	Synergy: 15% planned dam + gd26bi70 + synergistic effect	Each planned dam constructed by 15% probability	10500	614	GFDL−ESM2G	2.6	2070
292	Synergy: 20% planned dam + cc45bi70 + synergistic effect	Each planned dam constructed by 20% probability	10220	621	CCSM4	4.5	2070
293	Synergy: 25% planned dam + mc60bi50 + synergistic effect	Each planned dam constructed by 25% probability	11476	622	MIROC5	6.0	2050
294	Synergy: 30% planned dam + bc45bi70 + synergistic effect	Each planned dam constructed by 30% probability	14631	631	BCC−CSM1−1	4.5	2070
295	Synergy: 35% planned dam + gs45bi50 + synergistic effect	Each planned dam constructed by 35% probability	15327	643	GISS−E2−R	4.5	2050
296	Synergy: 40% planned dam + mr26bi70 + synergistic effect	Each planned dam constructed by 40% probability	16190	639	MIROC−ESM	2.6	2070
297	Synergy: 45% planned dam + gf85bi70 + synergistic effect	Each planned dam constructed by 45% probability	15041	640	GFDL−CM3	8.5	2070
298	Synergy: 50% planned dam + gf45bi50 + synergistic effect	Each planned dam constructed by 50% probability	14568	658	GFDL−CM3	4.5	2050
299	Synergy: 55% planned dam + hg45bi70 + synergistic effect	Each planned dam constructed by 55% probability	25775	665	HadGEM2−CC	4.5	2070
300	Synergy: 60% planned dam + in85bi50 + synergistic effect	Each planned dam constructed by 60% probability	22626	670	INMCM4	8.5	2050
301	Synergy: 65% planned dam + hg85bi70 + synergistic effect	Each planned dam constructed by 65% probability	23905	671	HadGEM2−CC	8.5	2070
302	Synergy: 70% planned dam + he45bi70 + synergistic effect	Each planned dam constructed by 70% probability	17491	675	HadGEM2−ES	4.5	2070
303	Synergy: 75% planned dam + hd26bi50 + synergistic effect	Each planned dam constructed by 75% probability	22772	688	HadGEM2−AO	2.6	2050
304	Synergy: 80% planned dam + cc60bi70 + synergistic effect	Each planned dam constructed by 80% probability	27348	696	CCSM4	6.0	2070
305	Synergy: 85% planned dam + mc26bi50 + synergistic effect	Each planned dam constructed by 85% probability	22162	694	MIROC5	2.6	2050
306	Synergy: 90% planned dam + he85bi70 + synergistic effect	Each planned dam constructed by 90% probability	27436	706	HadGEM2−ES	8.5	2070
307	Synergy: 95% planned dam + cn26bi50 + synergistic effect	Each planned dam constructed by 95% probability	26716	712	CNRM−CM5	2.6	2050
308	Synergy: 100% planned dam + ce45bi70 + synergistic effect	Each planned dam constructed by 100% probability	29027	717	CESM1−CAM5−1−FV2	4.5	2070
309	Synergy: 5% mainstream dam + he60bi50 + synergistic effect	Each planned lower Mekong mainstream dam constructed by 5% probability	9472	597	HadGEM2−ES	6.0	2050
310	Synergy: 10% mainstream dam + hd60bi50 + synergistic effect	Each planned lower Mekong mainstream dam constructed by 10% probability	9041	598	HadGEM2−AO	6.0	2050
311	Synergy: 15% mainstream dam + ip85bi70 + synergistic effect	Each planned lower Mekong mainstream dam constructed by 15% probability	8832	597	IPSL−CM5A−LR	8.5	2070
312	Synergy: 20% mainstream dam + he85bi50 + synergistic effect	Each planned lower Mekong mainstream dam constructed by 20% probability	9377	599	HadGEM2−ES	8.5	2050
313	Synergy: 25% mainstream dam + mg45bi70 + synergistic effect	Each planned lower Mekong mainstream dam constructed by 25% probability	9769	598	MRI−CGCM3	4.5	2070
314	Synergy: 30% mainstream dam + ac45bi70 + synergistic effect	Each planned lower Mekong mainstream dam constructed by 30% probability	16401	601	ACCESS1−0	4.5	2070
315	Synergy: 35% mainstream dam + mc60bi70 + synergistic	Each planned lower Mekong mainstream dam constructed by 35% probability	12349	600	MIROC5	6.0	2070
316	Synergy: 40% mainstream dam + gd60bi50 + synergistic effect	Each planned lower Mekong mainstream dam constructed by 40% probability	13391	599	GFDL−ESM2G	6.0	2050
317	Synergy: 45% mainstream dam + no85bi70 + synergistic effect	Each planned lower Mekong mainstream dam constructed by 45% probability	13763	603	NorESM1−M	8.5	2070
318	Synergy: 50% mainstream dam + ip60bi70 + synergistic effect	Each planned lower Mekong mainstream dam constructed by 50% probability	18265	605	IPSL−CM5A−LR	6.0	2070
319	Synergy: 55% mainstream dam + mp85bi70 + synergistic effect	Each planned lower Mekong mainstream dam constructed by 55% probability	17092	602	MPI−ESM−LR	8.5	2070
320	Synergy: 60% mainstream dam + mc85bi50 + synergistic	Each planned lower Mekong mainstream dam constructed by 60% probability	14098	602	MIROC5	8.5	2050
321	Synergy: 65% mainstream dam + mg26bi70 + synergistic effect	Each planned lower Mekong mainstream dam constructed by 65% probability	15828	603	MRI−CGCM3	2.6	2070
322	Synergy: 70% mainstream dam + mr60bi70 + synergistic effect	Each planned lower Mekong mainstream dam constructed by 70% probability	18800	606	MIROC−ESM	6.0	2070
323	Synergy: 75% mainstream dam + ip26bi70 + synergistic effect	Each planned lower Mekong mainstream dam constructed by 75% probability	20154	606	IPSL−CM5A−LR	2.6	2070
324	Synergy: 80% mainstream dam + ip85bi50 + synergistic effect	Each planned lower Mekong mainstream dam constructed by 80% probability	20333	607	IPSL−CM5A−LR	8.5	2050
325	Synergy: 85% mainstream dam + mc45bi70 + synergistic	Each planned lower Mekong mainstream dam constructed by 85% probability	18923	606	MIROC5	4.5	2070
326	Synergy: 90% mainstream dam + hg45bi50 + synergistic effect	Each planned lower Mekong mainstream dam constructed by 90% probability	20480	608	HadGEM2−CC	4.5	2050
327	Synergy: 95% mainstream dam + mg60bi50 + synergistic effect	Each planned lower Mekong mainstream dam constructed by 95% probability	21735	608	MRI−CGCM3	6.0	2050
328	Synergy: 100% mainstream dam + mp45bi50 + synergistic	Each planned lower Mekong mainstream dam constructed by 100% probability	21890	609	MPI−ESM−LR	4.5	2050
329	Synergistic: 5% tributary dam + mi85bi50 + synergistic effect	Each planned non−Mekong mainstream dam constructed by 5% probability	7966	601	MIROC−ESM−CHEM	8.5	2050
330	Synergistic: 10% tributary dam + mg85bi50 + synergistic effect	Each planned non−Mekong mainstream dam constructed by 10% probability	8121	611	MRI−CGCM3	8.5	2050
331	Synergistic: 15% tributary dam + no60bi70 + synergistic effect	Each planned non−Mekong mainstream dam constructed by 15% probability	8509	613	NorESM1−M	6.0	2070
332	Synergistic: 20% tributary dam + gf45bi70 + synergistic effect	Each planned non−Mekong mainstream dam constructed by 20% probability	8473	616	GFDL−CM3	4.5	2070
333	Synergistic: 25% tributary dam + he26bi70 + synergistic effect	Each planned non−Mekong mainstream dam constructed by 25% probability	9595	629	HadGEM2−ES	2.6	2070
334	Synergistic: 30% tributary dam + mi60bi70 + synergistic effect	Each planned non−Mekong mainstream dam constructed by 30% probability	10157	633	MIROC−ESM−CHEM	6.0	2070
335	Synergistic: 35% tributary dam + no26bi70 + synergistic effect	Each planned non−Mekong mainstream dam constructed by 35% probability	10125	635	NorESM1−M	2.6	2070
336	Synergistic: 40% tributary dam + he45bi50 + synergistic effect	Each planned non−Mekong mainstream dam constructed by 40% probability	10460	647	HadGEM2−ES	4.5	2050
337	Synergistic: 45% tributary dam + mg85bi70 + synergistic effect	Each planned non−Mekong mainstream dam constructed by 45% probability	11091	644	MRI−CGCM3	8.5	2070
338	Synergistic: 50% tributary dam + gd60bi70 + synergistic effect	Each planned non−Mekong mainstream dam constructed by 50% probability	11543	653	GFDL−ESM2G	6.0	2070
339	Synergistic: 55% tributary dam + cc85bi70 + synergistic effect	Each planned non−Mekong mainstream dam constructed by 55% probability	12296	657	CCSM4	8.5	2070
340	Synergistic: 60% tributary dam + no60bi50 + synergistic effect	Each planned non−Mekong mainstream dam constructed by 60% probability	12493	666	NorESM1−M	6.0	2050
341	Synergistic: 65% tributary dam + hd26bi70 + synergistic effect	Each planned non−Mekong mainstream dam constructed by 65% probability	12162	667	HadGEM2−AO	2.6	2070
342	Synergistic: 70% tributary dam + he60bi70 + synergistic effect	Each planned non−Mekong mainstream dam constructed by 70% probability	12417	674	HadGEM2−ES	6.0	2070
343	Synergistic: 75% tributary dam + bc26bi70 + synergistic effect	Each planned non−Mekong mainstream dam constructed by 75% probability	13666	674	BCC−CSM1−1	2.6	2070
344	Synergistic: 80% tributary dam + mr85bi70 + synergistic effect	Each planned non−Mekong mainstream dam constructed by 80% probability	13622	679	MIROC−ESM	8.5	2070
345	Synergistic: 85% tributary dam + cc45bi50 + synergistic effect	Each planned non−Mekong mainstream dam constructed by 85% probability	14076	687	CCSM4	4.5	2050
346	Synergistic: 90% tributary dam + gs85bi50 + synergistic effect	Each planned non−Mekong mainstream dam constructed by 90% probability	14384	691	GISS−E2−R	8.5	2050
347	Synergistic: 95% tributary dam + cc26bi70 + synergistic effect	Each planned non−Mekong mainstream dam constructed by 95% probability	14540	700	CCSM4	2.6	2070
348	Synergistic: 100% tributary dam + gf85bi50 + synergistic	Each planned non−Mekong mainstream dam constructed by 100% probability	14738	704	GFDL−CM3	8.5	2050
349	Synergistic: 5% dam removal + mc85bi70 + synergistic effect	Each existing dam removed by 5% probability	7522	584	MIROC5	8.5	2070
350	Synergistic: 10% dam removal + mg45bi50 + synergistic effect	Each existing dam removed by 10% probability	6825	525	MRI−CGCM3	4.5	2050
351	Synergistic: 15% dam removal + ip45bi70 + synergistic effect	Each existing dam removed by 15% probability	6526	494	IPSL−CM5A−LR	4.5	2070
352	Synergistic: 20% dam removal + mc45bi50 + synergistic effect	Each existing dam removed by 20% probability	5189	468	MIROC5	4.5	2050
353	Synergistic: 25% dam removal + no85bi50 + synergistic effect	Each existing dam removed by 25% probability	5852	460	NorESM1−M	8.5	2050
354	Synergistic: 30% dam removal + mg26bi50 + synergistic effect	Each existing dam removed by 30% probability	5354	403	MRI−CGCM3	2.6	2050
355	Synergistic: 35% dam removal + hd45bi50 + synergistic effect	Each existing dam removed by 35% probability	4389	383	HadGEM2−AO	4.5	2050
356	Synergistic: 40% dam removal + in45bi70 + synergistic effect	Each existing dam removed by 40% probability	3899	362	INMCM4	4.5	2070
357	Synergistic: 45% dam removal + ac45bi50 + synergistic effect	Each existing dam removed by 45% probability	4408	336	ACCESS1−0	4.5	2050
358	Synergistic: 50% dam removal + mp85bi70 + synergistic effect	Each existing dam removed by 50% probability	3241	287	MPI−ESM−LR	8.5	2070
359	Synergistic: 55% dam removal + gs26bi50 + synergistic effect	Each existing dam removed by 55% probability	4344	371	GISS−E2−R	2.6	2050
360	Synergistic: 60% dam removal + he45bi70 + synergistic effect	Each existing dam removed by 60% probability	2508	240	HadGEM2−ES	4.5	2070
361	Synergistic: 65% dam removal + gd45bi50 + synergistic effect	Each existing dam removed by 65% probability	2390	230	GFDL−ESM2G	4.5	2050
362	Synergistic: 70% dam removal + mr85bi50 + synergistic effect	Each existing dam removed by 70% probability	1933	158	MIROC−ESM	8.5	2050
363	Synergistic: 75% dam removal + bc26bi70 + synergistic effect	Each existing dam removed by 75% probability	1700	142	BCC−CSM1−1	2.6	2070
364	Synergistic: 80% dam removal + he85bi70 + synergistic effect	Each existing dam removed by 80% probability	1540	108	HadGEM2−ES	8.5	2070
365	Synergistic: 85% dam removal + no45bi50 + synergistic effect	Each existing dam removed by 85% probability	871	91	NorESM1−M	4.5	2050
366	Synergistic: 90% dam removal + bc85bi70 + synergistic effect	Each existing dam removed by 90% probability	527	48	BCC−CSM1−1	8.5	2070
367	Synergistic: 95% dam removal + ip26bi50 + synergistic effect	Each existing dam removed by 95% probability	212	24	IPSL−CM5A−LR	2.6	2050
368	Synergistic: 100% dam removal + ce45bi50 + synergistic	Each existing dam removed by 100% probability	0	0	CESM1−CAM5−1−FV2	4.5	2050
